# Potential Aroma Chemical Fingerprint of Oxidised Coffee Note by HS-SPME-GC-MS and Machine Learning

**DOI:** 10.3390/foods11244083

**Published:** 2022-12-16

**Authors:** Giulia Strocchi, Eloisa Bagnulo, Manuela R. Ruosi, Giulia Ravaioli, Francesca Trapani, Carlo Bicchi, Gloria Pellegrino, Erica Liberto

**Affiliations:** 1 Dipartimento di Scienza e Tecnologia del Farmaco, Università degli Studi di Torino, 10125 Turin, Italy; 2 Luigi Lavazza S.p.A.,10156 Turin, Italy

**Keywords:** coffee volatilome, oxidative note, aroma fingerprint, HS-SPME-GC-MS/FPD, machine learning

## Abstract

This study examines the volatilome of good and oxidised coffee samples from two commercial coffee species (i.e., *Coffea arabica* (arabica) and *Coffea canephora* (robusta)) in different packagings (i.e., standard with aluminium barrier and Eco-caps) to define a fingerprint potentially describing their oxidised note, independently of origin and packaging. The study was carried out using HS-SPME-GC-MS/FPD in conjunction with a machine learning data processing. PCA and PLS-DA were used to extrapolate 25 volatiles (out of 147) indicative of oxidised coffees, and their behaviour was compared with literature data and critically discussed. An increase in four volatiles was observed in all oxidised samples tested, albeit to varying degrees depending on the blend and packaging: acetic and propionic acids (*pungent, acidic, rancid*), 1-H-pyrrole-2-carboxaldehyde (*musty*), and 5-(hydroxymethyl)-dihydro-2(3H)-furanone.

## 1. Introduction

The quality of a coffee is related to the aroma and flavour developed because of the chemical reactions that take place during roasting and on the basis of the precursors present in the bean, which are due to various terroir and processing factors. The pleasant aroma of a freshly opened packet of roasted coffee is an additional indicator of quality [1]. Coffee is considered a stable product with a long shelf life, although it is still an active “chemical reactor” after roasting, as the components resulting from the pyrolysis of the nonvolatile precursors, the degradation of lipids and sugars, and the Maillard reaction(s) are very reactive.

During storage, chemical and physical changes can affect the quality of roasted coffee. The changes in sensory features are generally due to the loss of key aroma compounds and the appearance of oxidation products that can cause unpleasant flavours [2,3,4]. The loss of aroma freshness during storage, known as “staling”, that is “*a sweet but unlovely flavour and aroma of roasted coffee which reflects the oxidization of many of the pleasant volatiles and the loss of others; a change in the flavour and the acid constituents causing a partial bland tone”* [5], mainly depends on the temperature, humidity, presence of oxygen, and barrier capacity of the packaging [3,6,7,8,9].

Although sensory analysis performed by a professional panel is the reference system for assessing coffee quality, several studies have been conducted with the aim of combining sensory and chemical analysis to define the freshness of a product through an objective analytical approach [10,11,12,13]. Available studies have principally identified a number of chemical markers associated with ageing, but most of them have focused on the loss of the main key odorants rather than the formation of new compounds [2,14,15,16,17,18,19].

In particular, low-molecular-weight sulphur-containing compounds, such as methanethiol (*sulphurous*, *putrid*) and 2-furfurylthiol (*roasty*, *coffeelike*) [2,19], and Strecker aldehydes and α-dicarbonyl compounds, such as propanal (*ethereal pungent*, *earthy*), 2-methyl propanal, 2- and 3-methyl butanal (*malty*), 2,3-butanedione, and 2,3-pentanedione (*buttery*, *pungent*) [3,8,17,20], have been indicated as possible reasons for the loss of freshness in the aroma (Table S1).

Although several volatile markers have been proposed as a means of monitoring changes in coffee freshness, the adoption of their absolute amounts can be questioned, as they are influenced by their initial concentrations, which in turn depend on variables such as the blend, degree of roasting, grinding, and other factors [16,21]. On the other hand, the use of indices calculated using the ratio between the quantities of selected diagnostic components in the headspace is a more robust technique that better reflects the changes taking place in the headspace. Several indices have been proposed as indicators of coffee freshness and, thus, as possible quality indicators. Table 1 lists some of the quality indicators (volatiles and indices) proposed so far, which correlate with the decrease in freshness of the aroma. For a more comprehensive list of quality indicators related to aroma decline over time and under the conditions studied, see Table S1. The 2-methylfuran/2-butanone (M/B) ratio is one of the best known. Its decrease over time can be correlated with the appearance of a sweet, but not pleasant, aroma [3,15,16,21].

Other proposed indices of coffee freshness are those involving sulphur compounds; however, only the 2-furfurylthiol/hexanal (FFT/HE) and, recently, dimethyl disulphide/methanethiol (DMDS/MeSH) indices were found to be useful as indicators for staling over long-term storage [20].

Despite their number, studies in this field have always focused on one or two compounds in the expression of coffee freshness loss, rather than on the synergism between the components responsible for the oxidised perception of the overall coffee volatilome [22]. Furthermore, due to the complexity and dynamics of the chemistry involved, coffee degradation studies have mainly been conducted on a single species, package, or condition [22]. Systematic studies in this direction on different species, packagings, and materials have been conducted only in recent years [7,20,23]. Therefore, defining the oxidation chemical footprint of coffee can be an objective valuable tool to be used in screening as a support to the sensory panel in testing new and more sustainable packaging. In these perspectives, machine learning tools play a key role in extracting and describing relevant information encrypted in complex data by using different algorithms and visual tools [24,25,26,27,28].

This study investigates the volatilome of good-quality coffee (from now “good” for short) and oxidised coffee (i.e., *Coffea arabica* (arabica) and *Coffea canephora* (robusta)) in different packagings (i.e., standard with aluminium barrier and Eco-caps) by combining HS-SPME-GC-MS/FPD with machine learning to define a potential fingerprint describing the oxidised note of roasted coffee.

## 2. Materials and Methods

### 2.1. Reference Standards and Solvents

Reference compounds for key odourant identity confirmation were either obtained from the library of standards of the authors’ laboratory or purchased from Merck (Milan, Italy). They are listed in Table S2 in the Supplementary Materials and marked with an asterisk. The homologous series of n-alkanes (from *n*-C9 to *n*-C25) for linear retention index (*I*^T^_S_) determination and solvents (cyclohexane and dibutyl phthalate), all HPLC grade, were all obtained from Merck (Merck, Milan, Italy).

### 2.2. Coffee Samples

Samples of the different blends and packagings of commercial roasted coffee were supplied by Luigi Lavazza S.p.A. (Lavazza, Turin, Italy).

Samples included 30 R&G (roasted and ground) coffees for moka preparation from three lots packed under vacuum in a multilayer film with an aluminium barrier (M samples). M samples consist of 100% Arabica from Central and South America. with balanced fruity and floral notes. A set of Eco-caps in a modified atmosphere for espresso coffee (5 caps) from different lots of different blends named B and P (100% arabica of different origins) and I (50/50 arabica and robusta) for a total of 30 samples was also included. I samples are top-quality arabica from South America and robusta from Africa and Southeast Asia with spicy notes. B is a mix from Central and South America and washed arabica from organic cultivation. P is a blend of Brazilian, Asian, and Central-South American arabica, with slight caramel and chocolate notes.

Part of the coffee samples was stored at room temperature; the other part was subjected to accelerated ageing under stressful storage conditions in an oven at 37 °C and 50% relative humidity. The samples were classified as good (G) and oxidised (OX) by a trained industrial sensory panel. The oxidised note was defined as the intensity of the smell/aroma attributable to rancid notes, walnut oil, peanut shell, old dried fruit, and “old” coffee, also often referred to as a cardboard note, for example, damp or closed/stale pizza box directly/indirectly perceived by the olfactory organ. The reference standard to define the oxidative note was a moka coffee that had been kept open for 4 months at room temperature.

### 2.3. Sampling Conditions

Automated HS-SPME sampling was run on a combi-PAL AOC 5000 autoinjector assembled on a GC–MS system (Shimadzu, Milan, Italy).

SPME fibres, polydimethylsiloxane/divinylbenzene (PDMS/DVB). df 65 µm–1 cm, were obtained from Supelco (Supelco, Bellefonte, PA, USA). Fibres were conditioned before use as recommended by the manufacturer.

The internal standard IS (*n*-C13) for peak response normalization was preloaded onto the SPME fibre by exposing the SPME fibre to the headspace of a 5 µL IS standard stock solution in dibutyl phthalate (1000 mg/L) for 20 min at 50 °C [29,30,31].

### 2.4. GC–MS/FPD Instrument Setup

The GC–MS system consisted of a Shimadzu QP-2010 (Shimadzu, Milan, Italy) operating in EI mode at 70 eV. The GC transfer line was set at 260 °C, and the ion source temperature at 200 °C. The scan range was set to *m/z* 35–350 with a scan speed of 666 amu/s. A SolGel-WAX column (100% polyethylene glycol) (30 m × 0.25 mm dc, 0.25 µm df) from Trajan (Trajan, Melbourne, Australia) was used. The carrier gas was helium, which was used at a constant flow rate of 1 mL/min. The oven temperature program was 40 °C (1 min) to 200 °C at 3 °C/min, then to 250 °C (3 min) at 10 °C/min. The FPD detector was set 260 °C. All other analysis conditions were those reported in the GC–MS paragraph.

Injections for linear retention index (*I*^T^_S_) determination were carried out using the combi-PAL AOC 5000 autosampler: injection mode split; split ratio, 1:20; volume, 1 µL; and temperature, 250 °C. Fibre thermal desorption was performed in splitless mode.

Data were acquired with a LabSolutions GCMS version 4.3. (Shimadzu, Milan, Italy). Aroma components sampled from the coffee headspace were either identified by comparing their calculated *I^T^*s and mass spectra with those of authentic samples or, tentatively, with those collected in in-house and/or commercial libraries (Wiley 7N and Nist 14 Mass Spectral Data) or reported in the literature. Table S2 reports the compounds identified with their experimental and literature *I^T^*s, mass spectral similarity, and, where available, reference standards evidenced with an asterisk.

### 2.5. Statistical Analysis

Principal component analysis (PCA), partial least squares discriminant analysis (PLS-DA), the Kruskal–Wallis test, and violin plots were performed using the XLSTAT software ver. 2021.2.1 Addinsoft (Addinsoft, New York, NY, USA). PCA was used to visualize information and sample clusters as a function of combinations of variables, while PLS-DA was used to extrapolate the variables of importance in discriminating the two classes of samples (good/oxidised). The Kruskal–Wallis test was used to evaluate the significance of the selected analytes. The heat map was created by gene-e (https://software.broadinstitute.org/GENE-E/) Accessed 27 July 2022.

## 3. Results and Discussion

The following sections address: (a) the chromatographic profiling of the volatile fraction of R&G coffee samples and the differences between the good and oxidised samples; (b) the extraction of the informative volatiles describing oxidised coffees; (c) a comparison with literature data on the loss of coffee freshness and staling, focusing on potent odourants; and (d) the determination of potential markers of oxidised coffee regardless of packaging and blend.

### 3.1. Chemical Profiling of Good and Oxidised Samples

**Figure 1:**
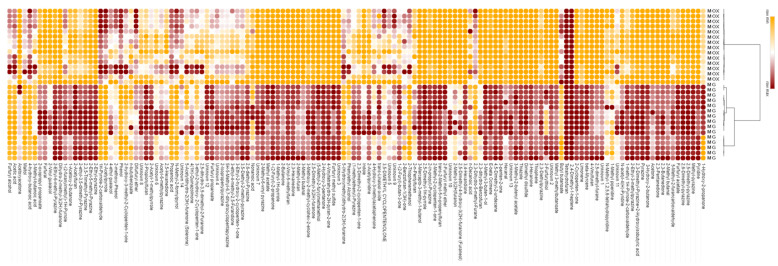
Heat map of the chemical normalized IS fingerprints of the good and oxidised moka samples (M). Samples are clustered using ascendant hierarchical clustering based on Euclidean distances using the average linkage agglomerative method. MG (moka good coffee) and MOX (moka oxidised coffees).

Although coffee is a stable product compared with other perishable foods, fresh aromas can be quickly lost over time. After roasting, coffee continues to be an active chemical reactor due to compounds formed from: (i) the pyrolysis of nonvolatile precursors (i.e., pyridine derivatives from trigonelline), (ii) the degradation of lipids and sugars (i.e., acrolein, dihydrofuranones, cyclopenten/exenolones, and pyrones), and (iii) the Maillard reaction (i.e., deoxyosone derivatives, 5-hydroxymethylfurfural, Strecker aldehydes, etc.) that are still highly reactive. Its stability over time can therefore also be influenced by the physical form of coffee (grains or powder), the barrier provided by the packaging, and external factors such as temperature, humidity, light, and the presence of oxygen. Appropriate profiling of the volatile fraction enables diagnostic and unbiased mapping of its volatilome [22]. In this study, volatile fraction sampling conditions and tools were set to achieve sufficient sensitivity to recover most of the volatiles describing the main aroma notes, while maintaining the complexity, and thus the informative power, of the coffee volatilome [30,31,32]. The analysed coffee samples were described by the 147 volatiles listed in Table S2. Figure 1 is an illustrative pattern of the chemical signatures of the good and oxidised moka samples (M) visualized with a heat map of the normalized volatile responses *versus* the IS (*n*-C13). Samples are clustered using ascendant hierarchical clustering based on Euclidean distances using the average linkage agglomerative method. The samples in the heat map are clustered into two groups: good (MG) and oxidised samples (MOX). A comparison of the two groups allows us to distinguish the pool of components with lower abundance (in orange), that is, those that are lost or degraded with the oxidation of coffee, from the components that are more abundant in the MOX samples (in brown), indicating their possible formation or increase with oxidation.

**Figure 2:**
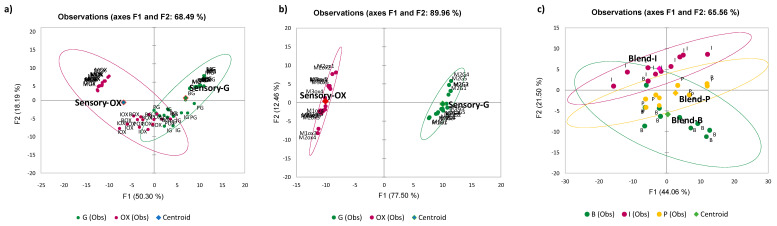
PCA of all samples investigated: (**a**) good and oxidised samples, 2, (**b**) moka good and oxidised samples, (**c**) blends in Eco-caps.

The principal component analysis (PCA) of all samples in Figure 2a displays two groups that are separated by the first PC component (F1). The clusters partially overlap because they include different blends, packagings (Eco and standard pack for M), and samples for different brewing modes (moka and caps for espresso coffee). Looking at the most standardised samples, that is, moka (Figure 2b), the clustering of oxidised and good samples is well defined for the first PC with an explained variance (expl. var.) of 77.50% compared with 50.30% of F1 in Figure 2a; this is nevertheless a positive result, indicating that the effect of ageing and oxidation is also when looking at different coffees and packs. The PCA carried out only on the cap samples shows the differences between the blends (Figure 2c); blends P and B are 100% arabica, while blend I is a 50/50 arabica and robusta mix. They also vary in the roasting processes to obtain the desired aroma for the different commercial blends.

### 3.2. Extraction of the Informative Volatiles of the Oxidised Note

Supervised PLS-DA machine learning was applied to the whole sample set to extract informative volatiles that describe the oxidised coffee note. The samples were split in a training set (*n* = 45) and a validation set (*n* = 15). The training set model was cross-validated by jackknife (CV = 5) and the model recomputing after removing each group from the training set, one by one. The cumulative Q^2^ statistics was 0.886 on the first two components with a classification rate of 93.33%.

Table 2 lists the 71 significant volatiles, out 147, with a VIP (variable importance in projection) > 1 in describing the oxidised samples. VIP scores estimate the importance of variables in the projection used in the PLS-DA model and are often used to select variables; when a variable presents a VIP that is close to or greater than 1, it can be considered important in the given model. Table 2 also reports the VIP standard deviations and boundaries at a 95% confidence limit, the Pearson correlation coefficient (r), and descriptions of odour quality.

Most volatiles present high r, and volatiles with an r > 0.7 are strongly correlated with oxidation. The components with 0.5 < r > 0.7 are moderately correlated and are more linked to the packaging. These data show that several volatiles are more abundant in oxidised samples, and some, in bold, are common to all packagings. Although coffee aroma is the result of the synergistic effect between several volatiles, some of the substances that correlate better with oxidation have been described as having an unpleasant odour.

This result means that several components change independently of the packaging and blends, although to different extents. Figure S1 shows the percentage coefficients of variation (CV%) of the normalized responses of the good *versus* oxidised samples in the different packages. The figure shows variations above 20%, which is the relative standard deviation of the analytical method (RSD% > 20), whose limits are indicated in the figure by the orange horizontal dashed lines. The CV% was calculated using Equation (1):

CV% = −[(good norm. response − oxidised norm. response)/good norm. response] × 100(1)

Moka samples (Figure S1a) show a significant reduction in aroma components compared with the capsules (Figure S1b–d), especially in the volatile components associated with freshness notes, such as sulphurous compounds (thiols), pyrazines, pyrroles, and alcohols; and this fact could be related to the higher surface exposure of the matrix to potential oxidation and humidity effects [33]. On the other hand, the composition of the volatile fraction of coffee in capsules varies less, probably because it is packaged under a modified atmosphere.

### 3.3. Comparison of the Informative Volatiles in Oxidised Coffees and Literature Data on Markers of Coffee Ageing

Several studies have investigated the change in coffee aroma over time, and most have focused on the loss of freshness (i.e., staling) and/or have monitored the evolution of aroma over a short period [34], with only a few investigating long-term effects [7,20]. Table S1 lists the volatiles that have been reported in previous studies as being linked to the deterioration of roasted coffee aroma.

Few volatiles characterising the oxidised coffees in our samples have already been described in the literature as ageing markers. The components that showed similar trends in the aged samples to those previously reported are: 2/3-methylbutanal (*malty*); 2,3-butandione (*buttery, pungent*); 2,3-pentandione (*buttery, pungent*); 2-furfurylthiol (*roasty, coffeelike*); 2,5-dimethyl-3(2H)-furanone (*caramellic*), 2,5-dimethyl-4-hydroxy-3(2H)-furanone (furaneol) (*sweet, candy*) (those with a VIP higher than 1 are highlighted with an asterisk in Table 2).

In this study, a number of components known to be related to ageing behaved differently in oxidised samples than described in the literature (i.e., 2-butanone, 2-methylfuran, hexanal, 2-acetylfuran, dihydro-2-methyl-3(2H)-furanone) (highlighted in italics in Table 2) or do not vary significantly (CV% below 20%) in all samples studied (i.e., 1-methyl-1H-pyrrole, N-acetyl-4(H)-pyridine). The latter observation, in particular, may be related to the different blends and packagings, here considered different from the literature data, indicating that staling or ageing indices depend on species, blend, and packaging [2,11,17,20].

For example, 2-butanone (*ethereal*) was observed to be present in lower amounts in oxidised samples M and I, as reported by Glöss and Marin, who noted a reduction over time [3,20,34], and on the opposite in B and P samples.

**Figure 3:**
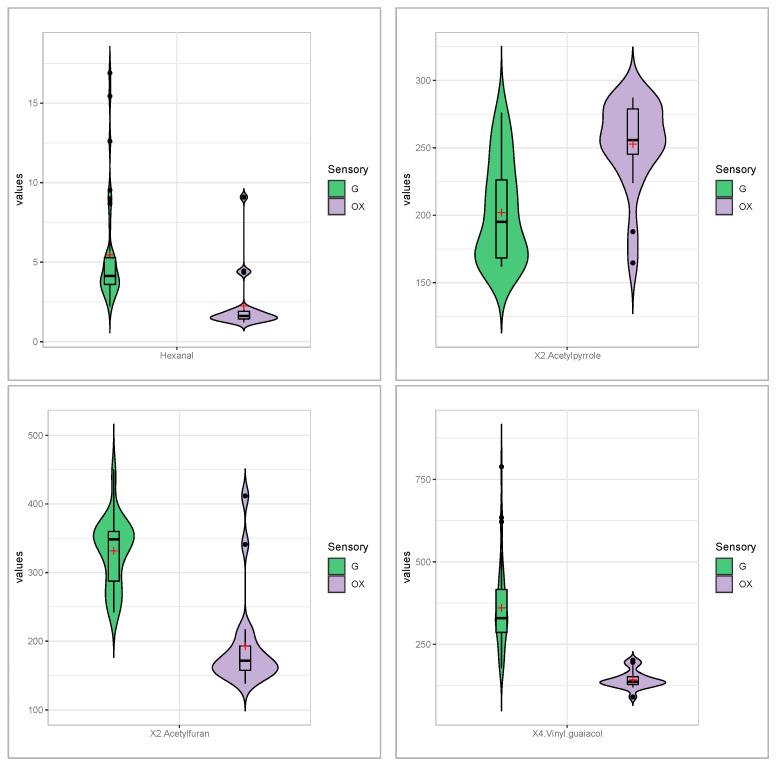
Violin plots of the trends of some markers from the literature in analysed samples. (+) represents the mean.

Hexanal (*fatty, sweaty*) is a known by-product and marker for the degradation of linoleic acid by autoxidation, which increased over time (Table S1) [3,14,35]. Surprisingly, it is present in lower amounts in the oxidised coffees than in the good coffees in our experiments (Figure 3). This can be explained by its high reactivity and the strongly oxidative environment, which leads to the formation of hexanoic acid (*sour fatty sweat*). 2-Acetylfuran (*sweet balsam, caramel*) decreased in amount in the M (100% arabica for moka) and I samples (a blend containing robusta in Eco caps); on the other hand, it increased in B and P (100% arabica in Eco caps) (Figure 3 and Table 2). The behaviour of 2-acetylfuran in I samples confirms the previous results of Cincotta et al., which cover 6 months of storage [7]. These authors also report that 2-acetylpyrrole (*musty, nutty*) decreases in blends containing robusta, while, in this study, it increased in aged samples (Figure 3 and Table S2). 4-Vinylguaiacol (*phenolic, smoky*) was indicated as a staling component by Kallio and Holscher [15,16] as it decreases with time, as it does in all oxidised coffees in these experiments, although its VIP is below 1 (i.e., 0.8919) and r is −0.5637 (Figure 3 and Table S2). This behaviour, however, is not confirmed in robusta samples in caps after 6 months’ storage [7] (Table S1).

Furfurylthiol (FFT—*roasty, coffeelike*) is known as a potent coffee odourant and freshness marker [19,32,35]. It is not included in the selected VIP because it is outside the fixed VIP cut-off. FFT has a VIP of 0.9713 and a moderate correlation with the oxidation process (r = 0.6323) and decreases dramatically in oxidised samples from the first stages of storage. The decrease in FFT may also be due to evaporation, adsorption onto the solid matrix, and internal reactions. FFT and all thiols in general are highly reactive nucleophiles that can degrade in the presence of hydroperoxides, oxidise (e.g., MeSH to DMDS), and react with phenolic compounds [36]. In particular, Hofmann et al. showed that FFT decreases because it becomes trapped by low-molecular-weight melanoidins, unlike what happens with mercaptan-aldehydes [37,38,39]. More recently, Glöss et al. [20] identified the ratio between DMDS (*sulphur, cabbage*) and MeSH (*sulphur, cabbage*) as a freshness index for coffee, and underlined that DMDS increases because of the oxidation of MeSH. This index was recognized by monitoring the transformation of coffee aroma quality over time at room temperature. This condition is not often investigated over a longer period of time, since the temporal evolution of coffee aroma, for example, to evaluate its shelf-life, is usually determined under accelerated conditions due to the stability of coffee over time. In the present study, this behaviour was observed in oxidised Eco-cap coffees, while the index was not measurable in M samples, since DMDS and DMTS (dimethyl trisulphide) (*onionlike*) were not detected. On the other hand, dimethyl sulphone (*sulphurous burnt*), which is a MeSH derivative that is related to strong oxidation, increased threefold (Table 3) [35].

### 3.4. Potential Markers of the Oxidised Coffee Note

Twenty-five highly significant volatiles describing oxidised coffees with a similar trend in all blends and packages studied, and with a CV% of at least 20, were identified as potential markers for oxidised coffees. They are listed in bold in Table 2.

Most of them are present in oxidised samples in lower amounts, some are already known to decrease over time (in italics in Table 2), and others behave differently than in previous studies. These compounds are highly reactive heterocycles, especially in the presence of humidity and oxygen, which could explain their decrease in oxidised coffees [35]. Four volatile compounds increased in all samples examined, albeit to varying degrees depending on the mixture and packaging: acetic and propionic acids (*pungent, sour, rancid*), 1-H-pyrrole-2-carboxaldehyde (*musty*), and 5-(hydroxymethyl)-dihydro-2(3H)-furanone.

Short-chain fatty acids are formed in roasted coffee by the breakdown of polysaccharides during roasting. Their continuous increase during storage could be due to the chemical cleavage of triglycerides (TGA), which contributes to the change in sensory properties [9].

The typical oxidised note perceived by the olfactory organ is due to the altered balance of the volatiles in oxidised samples and can be associated with rancid, walnut oil, peanut shell, old dried fruit, and “old” coffee notes. The coffee volatilome includes both odourant and nonodourant compounds, the latter of which contribute to a synergistic effect in aroma perception [40,41,42]. It has highly informative power in describing the potential evolution of aroma over time [7,15,21], and is a diagnostic tool for the “identitation” of blends/origins and the detection of applied technological processes [43,44,45,46].

## 4. Conclusions

Twenty-five target components of the coffee volatilome were identified as markers of coffee oxidation because they showed the same behaviour and statistical significance in all samples studied, regardless of packaging and blending. Together, they play a synergistic role in the detection of oxidised coffee and can be considered the fingerprint of the oxidised note. Four volatiles associated with ageing increased in all the packages studied: acetic and propionic acids (*pungent, sour, rancid*), 1-H-pyrrole-2-carboxaldehyde (*musty*), and 5-(hydroxymethyl)-dihydro-2(3H)-furanone, while the other 21 decreased in oxidised coffees.

The literature survey suggests several compounds as markers for the decrease in aroma quality of R&G coffee during storage, but they are not standardised. However, most of the proposed markers and indices seem to be more closely related to coffee aroma freshness, in particular: 2 and 3-methylbutanal (*malty*), 2-propanal (*ethereal pungent, earthy*), 2,3-butan and pentandione (*buttery, pungent*), dimethyl sulphide (cabbagelike), DMDS (*sulphur, cabbage*), MeSH (*sulphur, cabbage*), 2-FFT (*roasty, coffeelike*), and the ratios of 2-methylfuran/2-butanone, 2-methylfuran/2,3-butanedione, and MeSH/hexanal, the decrease of which was associated with coffee staling, or DMDS/MeSH, which increased with storage.

The composition of the volatilome also depends on the packaging and blend. A comparison of these results with those in the literature shows that some markers are in common and present in lower amounts in oxidised coffees; these include 2,3-butanedione; 2,3-pentandione; 2-furfurylthiol; acetic acid (*pungent, sour*) and furaneol (*sweet, candy*); and the indices 2-methylfuran/2-butanone and 2-butanone/2-methylfuran. Conversely, other components seem to be specific to the packaging and/or blend, including 2-methylfuran, 2-methylbutanal, 2-acetylfuran, 2,5-dimethylfuran (*meaty*), and 2-methyl-2-cyclopenten-1-one.

These data provide information on the oxidised note and show that artificial intelligence can be used successfully to instrumentally define the change in the quality of the coffee aroma over time.

## Figures and Tables

**Table 1:** Indices of coffee ageing from a literature survey, data adapted from references column.

Compound Ratio (Indices)	Trend over Time	Reference
Acetone/2,3-butanedione	Increase	[16,21]
2-Butanone/2-methylfuran	Increase	[20]
2-Methylfuran/methanol (M/M)	Increase	[3,5,16,21]
2,3-Butanedione/2-methylfuran	Increase	[16,20,21]
Dimethyl disulphide/methanethiol	Increase	[20]
2-Butanone/methanethiol	Increase	[20]
Acetone/propanal	Increase	[16,17,18,19,20,21]
Thiophene/propanal	Increase	[16,21]
Thiophene/2,3-butanedione	Increase	[16,21]
2-Methyl butanal/2,3-butandione	Increase	[16]
2-Methyl butanal/propanal	Increase	[16]
2-Methylfuran/2-butanone (M/B)	Decrease	[3,16,21]
2-Methylfuran/2,3-butanedione (M/BD)	Decrease	[3,16]
Methanethiol/hexanal (MT/HE)	Decrease	[3]
2,3-Butanedione/2-methylfuran	Decrease	[21]

**Table 2:** VIP from PLS-DA (variable important in the projection) > 1 in describing oxidised samples, volatile standard deviation and boundaries at a 95% confidence limit, Pearson correlation coefficient (r), and the description of the odour quality together with the compound CV% in the different blends/packagings, presented in conditional format, is reported (in yellow, CV% < −20, and in red, CV% > 20). Volatiles that decrease or increase in amount in all blends/packagings are indicated in bold; known volatiles from the literature that are related to staling are indicated in italics, while those that display different trends in comparison with literature data are indicated by *.

Variable	VIP	Standard Deviation	Lower Bound (95%)	Upper Bound (95%)	Pearson Corr Coeff (r)	Odour Description	M	B	P	I
**3-Penten-2-one**	1.4394	0.2165	1.0151	1.8637	**−0.9097**	**Sharp and acetone-like and fruity, phenolic and fishy**	**−100.00**	**−79.31**	**−77.15**	**−82.32**
**2-Methyl-6-vinyl pyrazine**	1.3987	0.1979	1.0108	1.7866	**−0.8840**	**Nutty**	**−73.05**	**−68.67**	**−66.57**	**−76.99**
**3-Mercapto-3-methyl-1-butanol**	1.3850	0.1752	1.0415	1.7284	**−0.8753**	**Meaty**	**−100.00**	**−43.63**	**−61.47**	**−94.05**
**1H-Pyrrole-2-carboxaldehyde**	1.3830	0.1574	1.0746	1.6914	**0.8741**	**Musty, corny, pungent**	**133.27**	**59.47**	**46.09**	**20.86**
Propanoic acid *	1.3826	0.1738	1.0420	1.7231	**0.8738**	**Pungent, sour milk**	**164.69**	**103.41**	**94.90**	**112.32**
**2-Cyclopenten-1-one**	1.3818	0.2082	0.9737	1.7898	**−0.8733**	**-**	**−80.82**	**−73.27**	**−72.19**	**−80.48**
2,5-Dimethyl-3(2H)-furanone *	1.3688	0.1657	1.0439	1.6936	**−0.8651**	**Caramellic**	**−83.62**	**−31.17**	**−31.05**	**−64.88**
4,5-Dimethyl-2-undecene	1.3621	0.1363	1.0949	1.6293	−0.8609	-	−100.00	−13.40	−30.64	−10.97
2,6-Dimethyl-pyrazine	1.3532	0.1186	1.1207	1.5857	−0.6007	chocolate	−83.13	−14.27	−14.93	−35.93
**Unknown 7 (m/z 94 Ti; 78; 137)**	1.3471	0.1335	1.0854	1.6088	**−0.8514**	**-**	**−93.79**	**−76.21**	**−63.80**	**−93.55**
2,5-Dimethyl-pyrazine	1.3441	0.1150	1.1187	1.5695	−0.8495	Nutty, peanut, musty, earthy	−81.37	−15.17	−12.90	−36.53
*trans*-2-Methyl-5-*n*-propenylfuran	1.3415	0.1059	1.1339	1.5491	−0.8479	-	−100.00	−6.93	−18.27	−27.64
(5-Methyl-2-furyl)methanethiol	1.3332	0.1208	1.0965	1.5700	−0.6007	Sulphurous roasted coffee	−93.81	9.07	−9.72	−58.88
2-Ethyl-pyrazine	1.3214	0.0768	1.1709	1.4719	−0.6007	Nutty	−86.00	−10.17	−6.47	−36.85
2,3-Dimethyl-pyrazine + 2-Hydroxyisobutyric acid	1.3167	0.0667	1.1860	1.4474	−0.8322	Nutty	−81.23	−21.05	−19.29	−35.15
3-Methyl-3-buten-1-ol	1.3157	0.1056	1.1086	1.5227	−0.8315	Sweet fruity	−100.00	2.27	−2.83	−45.65
β-Myrcene	1.3087	0.0987	1.1152	1.5022	−0.8271	Spicy	−93.49	−17.86	−39.94	−25.97
Unknown 2 (m/z 110 Ti; 67; 95; 110)	1.3063	0.0870	1.1357	1.4769	−0.8256	-	−100.00	-6.66	−10.90	−42.11
2-Hydroxy-3-pentanone	1.2972	0.0778	1.1447	1.4496	−0.8198	Truffle	−79.27	19.01	17.10	−19.21
1-(2-Furyl)-2-propanone	1.2963	0.0965	1.1071	1.4855	−0.8193	Caramellic fruity, spicy radish	−94.75	18.43	−2.16	−51.57
**2,3-Hexanedione**	1.2963	0.1139	1.0731	1.5195	**−0.8193**	**Buttery**	**−100.00**	**−44.12**	**−45.32**	**−81.16**
Methyl-pyrazine	1.2889	0.0497	1.1915	1.3864	−0.8146	Nutty	−88.20	1.20	8.18	−26.97
E-β Ocimene	1.2842	0.1056	1.0772	1.4911	−0.8116	Sweet herbal	−78.07	−18.08	−38.52	−25.83
**2,5-Dimethyl-1H-pyrrole**	1.2815	0.1200	1.0463	1.5167	**−0.8100**	**-**	**−100.00**	**−95.09**	**−91.86**	**−100.00**
**2-Vinyl-5-methylfuran**	1.2800	0.0953	1.0933	1.4667	**−0.8090**	**-**	**−99.62**	**−32.72**	**−33.07**	**−81.11**
2,3-Butanedione *	1.2706	0.1258	1.0240	1.5172	**−0.8030**	**Strong butter, sweet creamy, pungent caramel**	**−97.22**	**−70.20**	**−59.22**	**−74.93**
3-Methoxy-2-methyl-cyclohex-2-enone	1.2702	0.1347	1.0063	1.5341	−0.8028	-	−69.30	−13.64	−22.16	−42.05
**4-Vinyltetrahydro-2H-pyran-2-one**	1.2644	0.1275	1.0146	1.5142	**−0.7991**	**-**	**−98.02**	**−94.02**	**−82.92**	**−97.51**
**3,4-Hexandione**	1.2582	0.0860	1.0897	1.4267	**−0.7952**	**Buttery toasted, almond, nutty, caramellic**	**−100.00**	**−28.39**	**−33.45**	**−75.84**
Unknown 13 (m/z 57 Ti; 99; 149)	1.2499	0.0914	1.0708	1.4290	−0.7899	-	−100.00	−12.07	−21.41	−54.20
2-Ethyl-6-methyl-pyrazine	1.2489	0.0366	1.1771	1.3207	−0.7893	Nutty	−70.21	−19.38	−18.96	−26.23
**5-(Hydroxymethyl)-dihydro-2(3H)-furanone**	1.2486	0.1323	0.9893	1.5080	**0.7892**	**-**	**227.90**	**71.38**	**25.43**	**47.26**
N-Methyl-2-formylpyrrol	1.2421	0.1153	1.0162	1.4680	0.7850	Musty	59.35	34.05	34.82	−10.28
3-Hexanone	1.2420	0.0511	1.1418	1.3421	−0.7849	Fruity	−100.00	16.55	−2.18	−22.28
*1-Methyl-1H-pyrrole*	1.2304	0.0708	1.0916	1.3692	−0.7777	Powerful smoky woody	−99.27	−37.86	−15.68	−86.77
2-Furfuryl methyl ether	1.2298	0.0555	1.1210	1.3387	−0.7773	Roasted coffee	−100.00	4.02	−1.64	−25.91
Acetoxyacetone	1.2255	0.0553	1.1172	1.3338	−0.7745	Fruity buttery, dairy nutty	−56.87	4.86	7.71	−43.39
Methyl 3-methylbutanoate	1.2248	0.0373	1.1518	1.2978	−0.7741	Strong apple fruity	−100.00	−0.85	−8.82	−22.56
Unknown 3 (m/z 43 Ti; 71; 86)	1.2217	0.0650	1.0943	1.3491	−0.7705	-	−84.10	2.44	−3.15	−35.78
*Dihydro-2-methyl-3(2H)-furanone*	1.2191	0.0509	1.1194	1.3189	−0.7705	Bready	−96.01	18.00	19.12	−27.59
2-Ethyl-5-methyl-Pyrazine	1.2163	0.0542	1.1100	1.3226	−0.7687	Coffee bean	−65.21	−18.77	−18.27	−26.70
Acetic acid *	1.2122	0.1590	0.9005	1.5238	**0.7661**	**Sharp pungent sour vinegar**	**81.92**	**89.61**	**115.20**	**93.88**
2-Methyl-2-cyclopenten-1-one	1.1964	0.0822	1.0354	1.3574	−0.7562	-	−80.09	7.49	10.57	−29.86
**Unknown 12 (m/z 81 Ti; 53; 161)**	1.1908	0.0638	1.0657	1.3159	**−0.7526**	**-**	**−49.90**	**−31.58**	**−32.12**	**−75.80**
2-Acetylpyridine	1.1886	0.0713	1.0488	1.3284	−0.7512	Popcorn	−47.45	−0.70	−10.46	−32.11
2-Thiophenemethanol	1.1884	0.1027	0.9872	1.3897	0.7511	Savory roasted coffee	60.09	16.15	7.91	−24.96
3-Methyl-2-butenyl acetate	1.1855	0.0694	1.0496	1.3215	0.7511	Sweet fresh banana	−100.00	−16.60	−11.96	22.33
*2-Acetylfuran*	1.1743	0.1149	0.9491	1.3995	−0.7422	Sweet balsam, almond, cocoa, caramel coffee	−53.67	27.11	16.78	−17.42
Pyrazine	1.1702	0.1051	0.9642	1.3762	−0.7396	Pungent sweet corn like roasted hazelnut barley, floral	−93.84	18.49	37.49	-25.82
2,5-Dimethyl-4-hydroxy-3(2H)-furanone (Furaneol) *	1.1615	0.0986	0.9684	1.3547	**−0.7341**	**Sweet cotton candy caramel**	**−100.00**	**−51.51**	**−27.84**	**−79.86**
Unknown 1 (m/z 57 Ti; 43; 86)	1.1510	0.0689	1.0159	1.2861	−0.7275	-	−100.00	32.65	31.86	−15.13
Furfuryl methyl sulphide	1.1471	0.1065	0.9384	1.3558	−0.7250	Onion, garlic, sulphury	−100.00	4.89	−20.66	−58.08
Furfuryl acetate	1.1453	0.0570	1.0335	1.2571	−0.7239	Garlic, pungent vegetable, onion	−58.77	10.41	−6.46	−34.68
**2,3-dihydro-benzofuran**	1.1398	0.0967	0.9503	1.3292	**−0.7204**	**-**	**−69.61**	**−40.42**	**−55.80**	**−75.81**
2-Ethyl-3-methylpyrazine	1.1257	0.1581	0.8159	1.4356	−0.7115	Raw potato	−64.47	−19.53	−16.08	−20.59
**4-Vinylfuran**	1.1244	0.0512	1.0241	1.2247	**−0.7106**	**-**	**−100.00**	**−27.21**	**−29.47**	**−86.08**
3-Hydroxy-2-butanone	1.1204	0.1519	0.8227	1.4180	−0.7081	Sweet buttery creamy	−77.64	48.61	50.30	1.99
2,5-Dimethyl-furane	1.1092	0.0570	0.9975	1.2208	−0.7010	Meaty	−100.00	6.62	14.55	−36.44
***trans*-Linalool oxide**	1.1006	0.1842	0.7395	1.4617	**−0.6956**	**-**	**−20.50**	**−31.54**	**−43.72**	**−36.60**
1-Methyl 1H-Pyrrole-2-carboxaldehyde	1.0968	0.0968	0.9070	1.2866	−0.6932	-	−36.14	4.63	−1.09	−25.06
Limonene	1.0918	0.1373	0.8227	1.3609	−0.6900	Terpenic	−54.76	19.10	−45.30	−9.57
Pyridine	1.0895	0.1543	0.7871	1.3919	−0.6886	Fishy	−91.20	0.29	9.05	−29.83
Furfural *	1.0775	0.0508	0.9780	1.1770	**−0.6810**	**Sweet woody almond fragrant baked bread**	**−68.79**	**−38.45**	**−35.34**	**−64.13**
2-Propionylfuran	1.0759	0.1379	0.8056	1.3462	−0.6800	Fruity	−40.96	12.05	−2.07	−22.46
Butyl butanoate	1.0738	0.1853	0.7106	1.4370	0.6787	Sweet, fruity, fresh, diffusive, and ripe	178.85	38.13	−48.61	82.24
Thiazole	1.0715	0.2109	0.6580	1.4849	−0.6772	Fishy	−100.00	28.64	36.27	−4.72
N-acetyl-4(H)-pyridine	1.0695	0.1148	0.8444	1.2946	−0.6759	Burnt	−53.74	−16.85	−15.76	−48.11
Acetone	1.0676	0.2008	0.6740	1.4611	−0.6747	Ethereal, apple, pear	−97.27	21.50	98.39	−44.69
*2-Butanone*	1.0594	0.1815	0.7036	1.4151	−0.6583	Ethereal	−98.10	53.26	111.98	−11.23
2,3-Pentanedione *	1.0515	0.0663	0.9216	1.1814	**−0.6646**	**Buttery, sweet, nutty, pungent**	**−99.44**	**−54.20**	**−43.11**	**−79.92**
*2-Methyl-furan*	1.0416	0.0949	0.8556	1.2277	0.6054	Chocolate	−99.55	19.61	61.60	−52.88
3-Methyl butanal *	0.9974	0.0876	0.8257	1.1692	**−0.6304**	**Malty, cocoa, fruity**	**−99.37**	**−34.48**	**−21.75**	**−57.25**
2,3,5-Trimethyl-pyrazine	0.9929	0.2666	0.4703	1.5155	−0.6275	Nutty, cocoa, earthy	−50.68	−2.96	-3.57	−11.81
2,3-Dimethyl-2-cyclopenten-1-one	0.9904	0.2087	0.5813	1.3995	−0.6259	-	−33.81	−7.82	−14.35	−35.06
2-Furfurylthiol *	0.9713	0.0378	0.8973	1.0453	**−0.6139**	**Roasty (coffeelike)**	**−100.00**	**−100.00**	**−100.00**	**−100.00**
2-Methyl butanal *	0.9627	0.1193	0.7288	1.1966	−0.6084	Green, malty, buttery	−99.57	6.25	22.49	−11.14
1-Acetoxy-2-butanone	0.9613	0.1127	0.7404	1.1822	−0.6076	-	−43.87	−1.30	−7.75	−41.96
Indole	0.9600	0.1225	0.7200	1.2001	−0.6068	-	−52.19	−12.20	−20.45	−57.89

**Table 3:** Comparison of some sulphur markers from the literature and the DMDS/MeSH ratio in good and oxidised samples. ^§^ Under limit of detection.

	Dimethyl Sulphone		Dimethyl Trisulphide		Dimethyl Disulphide		Methanethiol		Ratio DMDS/MESH	
	Good	Ox	Good	Ox	Good	Ox	Good	Ox	Good	Ox
**M**	0.05	0.15	0.05	0.00	3.53	*n*.d.^§^	0.13	0.06	26.45	*n*.d. ^§^
**B**	0.07	0.03	0.07	0.03	2.65	5.61	0.19	0.08	14.01	74.25
**P**	0.07	0.07	0.10	0.02	3.18	19.09	0.16	0.12	19.74	153.94
**I**	0.06	0.10	0.10	0.04	3.50	12.44	0.11	0.12	30.85	100.02

## Data Availability

Data have been uploaded to the Open Science Framework (OSF) website in a dedicated repository: https://osf.io/uyv2b/. Accessed on 20 October 2022. The access is made available on request.

## Supplementary Materials

The following supporting information can be downloaded at: www.mdpi.com/article/10.3390/foods11244083/s1, Supplementary Table S1: List of volatile substances that have so far been associated in the literature with the deterioration of the aroma of roasted coffee. Supplementary Table S2: Volatiles identified with their experimental and literature retention indices (Its), target ion (Ti), and qualifier ions (Qis) and their mass spectral similarity index (SI). M, B, P, and I are the different packagings/blends for which the CV% of the coffee profiling, presented in conditional format, is reported (in yellow, CV% < −20, and in red, CV% > 20). * Volatiles confirmed by reference standard. Supplementary Figure S1: CV% graph visualization of the standardized responses of the chromatographic profiles together with the CV% cut-off > 20 (orange range).
