# Supplementary material for: Potential Aroma Chemical Fingerprint of Oxidised Coffee Note by HS-SPME-GC-MS and Machine Learning
Source: Foods. 2022 Dec 16;11(24):4083. doi: 10.3390/foods11244083 (PMC9778272; doi:10.3390/foods11244083)
Supplement: Supplementary file 1 [file foods-11-04083-s001.zip › Supplementary Tables.pdf]

# Potential Aroma Chemical Fingerprint Of Oxidised Coffee Note by HS-SPME-GC-MS and machine learning

Strocchi Giulia[a], Eloisa Bagnulo[a], Ruosi Manuela R.[b], Ravaioli Giulia[b], Trapani Francesca[b], Bicchi Carlo[a], Pellegrino Gloria[b], Liberto Erica[a]*

[a] Dipartimento di Scienza e Tecnologia del Farmaco, Università degli Studi di Torino, Via Pietro Giuria 9, Turin, Italy

[b] Luigi Lavazza S.p.A., Strada Settimo 410, Turin, Italy

*Corresponding author: erica.liberto@unito.it

**Table S1.** Lists the volatile substances that have so far been associated in the literature with the deterioration of the aroma of roasted coffee.

| Compounds | Trend over time | Aging conditions | Notes |
|---|---|---|---|
| **Indices** | | | |
| 2-Methylfuran and 2-butanone (M/B) | decrease | Linear decrease in air-permeable packages by about 0.1 unit a day [16]. Slower decrease in beans than in ground coffee samples; it remains constant for 7 months in beans and quickly decreases after grinding. Decrease during storage in dark roasted coffee[21]. | Component related to freshness. Decrease related to staling. Decrease of M/B index due to oxidation and loss of 2-methylfuran through diffusion [3,16]. |
| 2-Butanone/2-methylfuran | increase | Increase with storage in coffee beans packed in paper and plastic composite films; slight decrease in packages with aluminium layer [20] . | Increase related to staling. Ratio mainly driven by the high volatility of 2-methylfuran; increase with packaging material allowing 2-methylfuran to evaporate. With packaging preventing its loss, the ratio is mainly governed by the small differences in intrinsic reactivity among the two compounds. This ratio increases in absence of an aluminium layer in the packaging material leads; very small variations over time are observed with packaging with aluminium layer [20]. |
| 2-Methylfuran/ methanol (M/M) | increase | Increase in air-permeable bags during 12 weeks' storage [16] | Indicator of ageing [21].<br>Related to freshness [3,5] |
| | | | Freshness index [5,8] |
| 2-Methylfuran and 2,3-butanedione (M/BD) | decrease | Ratio decrease in coffee punctured packages [3,16] | Decrease related to staling [3] |
| 2,3-Butanedione / 2-methylfuran | increase | Increase with storage in coffees packed in paper and plastic composite films. Slight decrease in packages with aluminium layer [16,20-21] | Index evolution depends on packaging [20] |
| 2-Methylfuran and propanal (M/P) | | | Freshness index [3] |
| Methanethiol and 2,3-butanedione (MT/BD) | | | Freshness index [3] |
| Dimethyl disulphide/methanethiol | increase | Increase with storage in coffee capsules packed in aluminium layer package; stable with packages 100% aluminium [20] | Methanethiol is a highly volatile and reactive compound; it tends to oxidize and dimerize to dimethyl disulphide [20] |
| Methanethiol and hexanal (MT/HE) | decrease | | Freshness index [3] |
| 2-Butanone/methanethiol | increase | Increase with storage in coffee aluminium layered capsules, stable with 100% aluminium packages[20] | Methanethiol is a highly volatile and reactive compound; it tends to oxidize and dimerize to dimethyl disulphide [20] |

| | | | |
|---|---|---|---|
| Acetone / 2,3-butanedione | increase | | Increase with storage [16,21] |
| Acetone /propanal | increase | | Indicators of coffee ageing [16-21] |
| Thiophene/propanal | increase | | Indicators of coffee ageing [21]. Increase with storage [16,21] |
| Thiophene/ 2,3-butanedione | increase | | Indicator of coffee ageing; increase with storage [16,21] |
| 2,3-Butanedione/2-methylfuran | decrease | | Indicator of coffee ageing [21] |
| 2-Methyl butanal/2,3-butandione | increase | | Increase with storage [16] |
| 2-Methyl butanal/propanal | increase | | Increase with storage [16] |
| **Aldheydes** | | | |
| Hexanal | increase | Increase dependent on the time of exposure of roasted and ground coffee to air, in particular, with storage in air and in punctured packages [3]. More than double increase after 83 days of storage in valve bags under nitrogen atmosphere at 37°C (with less than 1% of oxygen) [14] | It is not a potent odorant, but its secondary oxidation volatile products develop oxidised off-notes in particular when the positive flavour compounds in roasted coffee start to decompose/evaporate [14]. Odour not characteristic of stale coffee. Not influenced by humidity [14] |
| Acetaldehyde | | At room temperature, a small decrease for pads individually packed and hermetically sealed than in pads that were enclosed together in a secondary pack [6]. The loss is less drastic when pads were stored in plastic bottles [6]. | |
| Propanal | decrease | Decrease by more than 80% in permeable bag [16,21] | |
| 2-Methylpropanal | decrease | At room temperature, a small decrease for pads individually packed and hermetically sealed than in pads that were enclosed together in a secondary pack [6]. Decrease by more than 50% after 74 days storage in permeable bag [16]. | Evaporation or degradation by oxidation [3]. Intense aromatic note in freshly roasted coffee [15]. |
| 2-Methylbutanal | decrease | At room temperature, a small decrease for pads individually packed and hermetically sealed than in pads that were enclosed together in a secondary pack [6]. Decrease by more than 50% after 74 days storage in permeable bag [16]. Degradation after 83 days of storage in valve bags at 37°C under nitrogen atmosphere (with less than 1% of oxygen with an oxygen concentration lower than 1%) [14]). | Quick degradation in coffees with high moisture content [14]. Evaporation or degradation by oxidation. Contribution to the freshness of roasted coffee aroma. Coffee ageing indicator [3,15] |
| 3-Methylbutanal | decrease | At room temperature, a small decrease for pads individually packed and hermetically sealed than in pads that were enclosed | Fast degradation in coffee with the highest moisture content [14]. Evaporation or degradation by oxidation. It contributes to |

| | | | |
|---|---|---|---|
| | | together in a secondary pack [6]. Decrease by more than 50% after 74 days storage in permeable bag [16] . | the freshness of roasted coffee aroma. Coffee ageing indicator [3,15]. |
| **ketones** | | | |
| 2,3-Butanedione | decrease | It significantly decreases in coffee packed under an air atmosphere than under a modified atmosphere [3]. At room temperature, a small decrease for pads individually packed and hermetically sealed than in pads that were enclosed together in an secondary pack [6]. Decrease by more than 50% after 74 days storage in permeable bag. Decrease of more than 80% in permeable bags [16].<br>Degradation after 83 days of storage at 37°C in valve bags under nitrogen atmosphere (with less than 1% of oxygen) [14]. | Associate with buttery aroma in ground and brewed coffee [3,15]. Fast degradation in coffees with high moisture content [14]. |
| 2-Butanone | decrease | | Indicator of roasted coffee staling and ageing. [21] |
| 2,3-Pentanedione | decrease | At room temperature, a small decrease for pads individually packed and hermetically sealed than in pads that were enclosed together in a secondary pack [6]. Decrease by more than 50% after 74 days storage in permeable bag [16]. Degradation after 83 days of storage at 37°C in valve bags under nitrogen atmosphere (with less than 1% of oxygen) [14]. | Associate with buttery flavour in ground coffee and coffee brew [3,15]. Degrades faster in the coffee with highest moisture content [14]. |
| **Sulphur compounds** | | | |
| Dimethylsulphide | decrease | Degradation after 83 days of storage at 37°C in valve bags under nitrogen atmosphere (with less than 1% of oxygen) [14] | Fast degradation in coffees with high moisture content [14]. It contributes to the freshness of roasted coffee [14] |
| Dimethyldisulphide | decrease | Absence of significant variations throughout storage in the brews at 4 °C. Progressive and significant decrease in brews stored at 25 °C [28]. Losses during the storage of roasted coffee beans [15] and ground roasted coffee [16] in air. | |
| 2-Furfurylthiol | decrease | Accelerated degradation in absence of protection from external conditions by proper packaging [3].<br>Significant reduction in coffee packed under air than in those under modified atmosphere [3]. Degradation after 83 days of storage at 37°C in valve bags under nitrogen atmosphere (with less than 1% of oxygen) [14] | Used as an indicator of coffee staling. Evaporation or degradation by oxidation [3]. Fast degradation with high moisture content [14]. |
| Methanethiol | decrease | It showed a decrease over time [15]. | Strong impact on aroma freshness [3]. Evaporation or degradation because of oxidation [3]. Its loss is correlated to the loss of freshness [5] |

| | | | |
|---|---|---|---|
| 2-Furan methanethiol | decrease | | It contributes to the pleasant aroma of fresh roasted coffee [15] |
| 3-Methyl-2-buten-1-thiol | decrease | | It contributes to the pleasant aroma of fresh roasted coffee [15] |
| Furfuryl mercaptan | increase | Increase during storage in permeable bags [16] | Responsible for stale flavor[5] |
| 5-Methylfurfuryl mercaptan | increase | Increase during storage in permeable bags [16] | Responsible for stale flavor[5] |
| Dimethyl trisulphide | increase | Increase at atmospheric pressure and under vacuum [14] | It derives from oxidation of methanethiol. Large increase with increasing of moisture content, related to a faster oxidation of thiols [14]. |
| Carbon disulphide | decrease | Decrease by more than 75% after 74 days storage in permeable bag [16] | |
| Thiophene | decrease | Decrease by more than 80% in permeable bag [16] | |
| Thiophene-3-aldehyde | decrease | Decrease by more than 80% in permeable bag [16] | |
| 3-Methylthiophene | decrease | Decrease by more than 80% in permeable bag [16] | |
| **Pyrazines** | | | |
| 2,6-Diethylpyrazine | increase [3] decrease [7] | It increases during storage in air and in punctured packages [3]. Decrease in Robusta coffee caps after 6 months of storage [7]. | Odour not characteristic of stale coffee. |
| 2,3-Diethylpyrazine | decrease | Decrease in Arabica coffee caps [7] | |
| 2-Ethylpyrazine | | No losses in two pads during storage due to its high boiling point (above 115°C) [6] | |
| 2-Acetyl pyrazine | decrease | Decrease in Arabica coffee caps [7]. Decrease in Robusta after 6 months of storage [7]. | |
| 2-Ethyl-3,5-dimethylpyrazine | decrease | Decrease in Robusta after 6 months of storage [7]. | |
| **Esters** | | | |
| Ethylacetate | decrease | Higher decrease in coffee packed under air than under modified atmosphere [3] | |
| Methyl acetate | decrease | Decrease by more than 80% in permeable bag [16] | |
| Methylthioacetate | decrease | Decrease by more than 80% in permeable bag [16] | |
| **Furans** | | | |

| | | | |
|---|---|---|---|
| 2-Furfurylfuran | decrease | Decrease in Robusta after 6 months of storage [7]. | |
| 2-Acetylfuran | decrease | No losses in two pads during storage due to its high boiling point (above 115°C) [6]. Decrease in Robusta coffee caps after 6 months of storage [7]. | |
| 2-Methylfuran | decrease | Decrease by more than 50% after 74 days storage in permeable bag [20] | Indicator of staling roasted coffee and aging [21]. Formed by pyrolysis, decrease in presence of oxygen [21]. |
| 2,5-Dimethylfuran | decrease | Decrease by more than 80% in permeable bag [16] | Indicator of the change of ground coffee [21] |
| 2-Ethylfuran | decrease | Decrease by more than 80% in permeable bag [16] | |
| Furfural | increase | | Oxidation product of furfuryl alcohol [8] |
| Methyl formate | decrease | Decrease by more than 80% in permeable bag [16] | |
| 3-Mercapto-3-methyl butyl formate | decrease | Degradation after 83 days of storage at 37°C in valve bags at nitrogen atmosphere (with an oxygen concentration lower than 1%) [14]. | Decrease more in coffee with high moisture content [14] |
| **Furanones** | | | |
| Dihydro-2- methyl-3(2H)-furanone | | No losses in two pads during storage due to the high boiling point (above 115°C) [6] | |
| 2,5-Dimethyl-2,3-dihydrofuranone | decrease | Decrease in Arabica coffee caps [7] | |
| Furaneol | decrease | Decrease in Arabica coffee caps [7] | |
| **Phenolic compounds** | | | |
| 4-Vinylguaiacol | increase [16] decrease [7] | Increase during storage in permeable bags [16]. Decrease in Robusta coffee caps after 6 months of storage [7]. | Responsible for stale flavor [15] |
| 4-Ethylguaiacol | decrease | Decrease in Robusta coffee caps after 6 months of storage [7]. | |
| Guaiacol | decrease | Decrease in Robusta coffee caps after 6 months of storage [7]. | |
| Phenol | decrease | Higher decrease in coffee packed under air than under modified atmosphere [3]. | |
| **Pyrroles** | | | |
| 1-Methylpyrrole | decrease | Higher decrease in coffee packed under air than under modified atmosphere [3]. Degradation after 83 days of storage at 37°C in valve bags under nitrogen atmosphere (with less than 1% of oxygen) [14]. | Quick degradation in coffee with high moisture content [14]. |
| 2-Acetylpyrrole | decrease | Decrease in Robusta caps after 6 months of storage [7]. | |
| 1-Furfurylpyrrole | decrease | Decrease in Robusta coffee caps after 6 months of storage [7]. | |

| | | | |
|---|---|---|---|
| 1-Furfuryl-2-methylpyrrole | increase | Increase during storage in permeable bags [16]. | Responsible for stale flavor |
| 2-Formyl-1-methylpyrrole | decrease | Decrease in Robusta coffee caps after 6 months of storage [7]. | |
| **Pyridines** | | | |
| N-acetyl-4H-pyridine | decrease | Decrease in Robusta coffee caps after 6 months of storage [7]. | |
| **Acids** | | | |
| Acetic acid | increase | Volatile fraction of Arabica caps, stable during storage up to 12 months. Increase of free fatty acids (FFA), similar behaviour in Robusta coffee caps [7]. | |
| Propionic acid | increase | Volatile fraction of Arabica caps, stable during storage up to 12 months. Increase of free fatty acids (FFA), similar behaviour in Robusta coffee caps [7]. | |
| Tetradecanoic acid | increase | The volatile fraction of Arabica coffee caps, remains quite stable during shelf life up to 12 months of storage. After this period free fatty acids (FFAs) increase even in Robusta coffee caps | |
| Pentadecanoic acid | increase | Volatile fraction of Arabica caps, stable during storage up to 12 months. After this period FFAs increase even in Robusta coffee caps [7]. | |
| Hexadecanoic acid | increase | Volatile fraction of Arabica caps, stable during storage up to 12 months. After this period, FFAs increase even in Robusta coffee caps [7]. | |
| 3-Methylbutanoic acid | | No losses in two pads during storage due to its high boiling point (above 115°C) [6]. | |

**Table S2.** *Volatiles identified with their experimental and literature retention indices ITs, Target ion (Ti) and qualifier ions (Qis) and their mass spectral similarity index (SI). M, B, P and I are the different packaging/blends for which the CV% of the coffee profiling, presented in conditional format, is reported (in yellow CV%<-20 and in red CV%>20). *Volatiles confirmed by reference standard.*

| N° | Compounds name | Rt | $I^T$calc | $I^T$ref | Ti | Qi | SI | M | B | P | I |
|---|---|---|---|---|---|---|---|---|---|---|---|
| 1 | Acetone* | 2.22 | 821 | 820 | 43 | 58-42 | 98 | -97.27 | 21.50 | 98.39 | -44.69 |
| 2 | Methyl acetate* | 2.30 | 832 | 825 | 43 | 74-42 | 94 | -100.00 | 31.45 | 103.40 | -59.67 |
| 3 | Tetrahydrofuran* | 2.48 | 854 | - | 42 | 72-58 | 97 | nd | -49.01 | -9.19 | 40.89 |
| 4 | 2-Methyl-furan | 2.62 | 871 | 868 | 82 | 53-81 | 98 | -99.55 | 19.61 | 61.60 | -52.88 |
| 5 | 2,4-Dimethyl-1-heptene | 2.68 | 885 | 885 | 43 | 55-70 | 96 | nd | nd | nd | nd |
| 6 | 2-Butanone* | 2.89 | 903 | 899 | 43 | 72-57 | 96 | -98.10 | 53.26 | 111.98 | -11.23 |
| 7 | 2,3-Dihydro-5-methyl-Furane | 2.95 | 907 | - | 84 | 39-83 | 80 | 95.04 | 23.62 | 22.36 | 5.75 |
| 8 | 2-Methyl butanal | 3.03 | 912 | 907 | 57 | 41-58 | 96 | -99.57 | 6.25 | 22.49 | -11.14 |
| 9 | 3-Methyl butanal | 3.08 | 916 | 925 | 44 | 41-43 | 97 | -99.37 | -34.48 | -21.75 | -57.25 |
| 10 | 2,5-dimethyl-furane | 3.58 | 958 | 958 | 96 | 81-53 | 97 | -100.00 | 6.62 | 14.55 | -36.44 |
| 11 | 1-Methyl piperidine | 3.11 | 959 | - | 98 | 57-43-71 | 96 | -62.27 | -88.10 | -95.87 | -90.86 |
| 12 | Unknown 1 (m/z 57; 43; 86; 41) | 3.92 | 971 | - | 57 | 43-86-41 | 89 | -100.00 | 32.65 | 31.86 | -15.13 |
| 13 | 2,3-Butanedione* | 3.99 | 977 | 998 | 43 | 86-42 | 99 | -97.22 | -70.20 | -59.22 | -74.93 |
| 14 | Methyl 3-methylbutanoate | 4.70 | 1043 | 1018 | 74 | 43-59-85 | 91 | -100.00 | -0.85 | -8.82 | -22.56 |
| 15 | Thiophene* | 4.79 | 1045 | 1022 | 84 | 58-45 | 93 | -100.00 | -41.94 | -52.01 | -56.38 |
| 16 | N-Methyl-1,2,5,6-tetrahydropyridine | 4.58 | 1039 | - | 96 | 54-82-68 | 95 | -61.23 | -87.97 | -95.17 | -85.11 |
| 17 | 3-Hexanone* | 5.39 | 1064 | 1052 | 57 | 43-71-100 | 93 | -100.00 | 16.55 | -2.18 | -22.28 |
| 18 | Unknown 2 (m/z 110; 67; 95) | 5.53 | 1068 | - | 110 | 67-95 | 91 | -100.00 | -6.66 | -10.90 | -42.11 |
| 19 | 2,3-Pentanedione* | 5.68 | 1073 | 1085 | 43 | 57-100 | 98 | -99.44 | -54.21 | -43.11 | -79.92 |
| 20 | Dimethyl disulphide* | 5.94 | 1081 | 1071 | 94 | 79-61 | 91 | -100.00 | 64.48 | 171.94 | 42.80 |
| 21 | 4-Vinylfuran | 6.05 | 1084 | - | 94 | 65-66-39 | 96 | -100.00 | -27.21 | -29.47 | -86.08 |
| 22 | Hexanal* | 6.19 | 1088 | 1084 | 56 | 72-44 | 93 | -62.17 | -63.17 | -54.61 | -3.67 |
| 23 | Unknown 2' (m/z 43; 57; 69) | 6.77 | 1106 | - | 43 | 57-69 | 66 | nd | nd | nd | nd |
| 24 | 3,3,5-Trimethyl-1,5-heptadiene | 6.63 | 1102 | - | 59 | 43-44-57 | 93 | nd | nd | nd | nd |
| 25 | 4,5-Dimethyl-2-undecene | 6.97 | 1112 | - | 69 | 41-95-70 | 95 | -100.00 | -13.40 | -30.64 | -10.97 |

| | | | | | | | | | | |
|---|---|---|---|---|---|---|---|---|---|---|
| 26 | 3-penten-2-one | 7.37 | 1125 | 1110 | 69 | 41-43-39 | 87 | -100.00 | -79.31 | -77.15 | -82.32 |
| 27 | 2,3-Hexanedione* | 7.67 | 1134 | 1136 | 43 | 71-41 | 93 | -100.00 | -44.12 | -45.32 | -81.16 |
| 28 | 1-Methyl-1H-pyrrole | 7.90 | 1141 | 1137 | 81 | 80-53 | 96 | -99.27 | -37.86 | -15.68 | -86.77 |
| 29 | 3,4-Hexandione | 7.94 | 1142 | 1151 | 57 | 114-58-56 | 98 | -100.00 | -28.39 | -33.45 | -75.84 |
| 30 | 2-Vinyl-5-methylfuran | 8.39 | 1156 | - | 108 | 107-43 | 95 | -99.62 | -32.72 | -33.07 | -81.11 |
| 31 | beta-Myrcene* | 8.70 | 1165 | 1157 | 93 | 41-69-91 | 93 | -93.49 | -17.86 | -39.94 | -25.97 |
| 32 | Pyridine* | 9.04 | 1176 | 1188 | 79 | 52-51-50 | 98 | -91.20 | 0.29 | 9.05 | -29.83 |
| 33 | Limonene* | 9.72 | 1197 | 1205 | 68 | 111-93-136 | 95 | -54.76 | 19.10 | -45.30 | -9.57 |
| 34 | Pyrazine* | 10.24 | 1211 | 1210 | 80 | 53-52 | 95 | -93.84 | 18.49 | 37.49 | -25.82 |
| 35 | Butyl butanoate | 10.14 | 1208 | 1212 | 71 | 89-56-43 | 84 | 178.85 | 38.13 | -48.61 | 82.24 |
| 36 | 2-n-Pentylfuran* | 11.16 | 1234 | 1230 | 81 | 138-94 | 91 | -27.13 | -39.79 | -63.85 | 95.69 |
| 37 | 2-Furfuryl methyl ether* | 11.46 | 1242 | 1243 | 81 | 53-112-82 | 91 | -100.00 | 4.02 | -1.64 | -25.91 |
| 38 | Thiazole* | 11.69 | 1248 | 1246 | 85 | 58-45-57 | 95 | -100.00 | 28.64 | 36.27 | -4.72 |
| 39 | 3-Methyl-3-buten-1-ol* | 11.80 | 1251 | 1245 | 56 | 86-41-68 | 92 | -100.00 | 2.27 | -2.83 | -45.65 |
| 40 | E-beta Ocimene | 11.92 | 1254 | 1250 | 93 | 91-136-105 | 89 | -78.07 | -18.08 | -38.52 | -25.83 |
| 41 | 3-Methyl-2-butenyl acetate | 11.85 | 1252 | 1251 | 43 | 86-68-53 | 90 | -100.00 | -16.60 | -11.96 | 22.33 |
| 42 | Methyl-pyrazine | 12.22 | 1262 | 1264 | 94 | 67-40 | 99 | -88.20 | 1.20 | 8.18 | -26.97 |
| 43 | Dihydro-2-methyl-3(2H)-furanone | 12.33 | 1265 | 1269 | 43 | 72-100-44 | 99 | -96.01 | 18.00 | 19.12 | -27.59 |
| 44 | 2,5-Dimethyl-1H-pyrrole | 12.56 | 1271 | - | 94 | 95-53 | 97 | -100.00 | -95.09 | -91.86 | -100.00 |
| 45 | 3-Hydroxy-2-butanone | 13.12 | 1285 | 1277 | 45 | 43-88 | 94 | -77.64 | 48.61 | 50.30 | 1.99 |
| 46 | trans-2-Methyl-5-n-propenylfuran | 13.39 | 1292 | 1267 | 122 | 79-43-121 | 94 | -100.00 | -6.93 | -18.27 | -27.64 |
| 47 | 1-Hydroxy-2-propanone* | 13.12 | 1285 | 1274 | 43 | 74-42 | 99 | -52.64 | 21.22 | 0.14 | -43.67 |
| 48 | 2,5-Dimethyl-pyrazine | 14.38 | 1317 | 1318 | 108 | 42-81 | 98 | -81.37 | -15.17 | -12.90 | -36.53 |
| 49 | 2,6-Dimethyl-pyrazine | 14.66 | 1324 | 1330 | 108 | 42-40 | 98 | -83.13 | -14.27 | -14.93 | -35.93 |
| 50 | 2-Ethyl-pyrazine | 14.94 | 1330 | 1334 | 107 | 108-80 | 99 | -86.00 | -10.17 | -6.47 | -36.85 |
| 51 | 2,3-Dimethyl-Pyrazine | 15.32 | 1340 | 1335 | 108 | 67-59 | 96 | -81.23 | -21.05 | -19.29 | -35.15 |
| 52 | 2-Cyclopenten-1-one* | 15.68 | 1349 | 1341 | 82 | 108-123 | 93 | -80.82 | -73.27 | -72.19 | -80.48 |
| 53 | Unknown 3 (m/z 43 Ti; 71; 86) | 15.87 | 1353 | - | 43 | 71-86 | 97 | -84.10 | 2.44 | -3.15 | -35.78 |
| 54 | 2-Hydroxy-3-pentanone | 16.08 | 1359 | 1361 | 45 | 57-58-84 | 98 | -79.27 | 19.01 | 17.10 | -19.21 |
| 55 | 2-Methyl-2-cyclopenten-1-one* | 16.21 | 1362 | 1367 | 67 | 96-53 | 97 | -80.09 | 7.49 | 10.57 | -29.86 |

| | | | | | | | | | | | |
|---|---|---|---|---|---|---|---|---|---|---|---|
| 56 | 2-Ethyl-6-methyl-Pyrazine | 17.00 | 1381 | 1375 | 121 | 122-94 | 99 | -70.21 | -19.38 | -18.96 | -26.23 |
| 57 | 2-Ethyl-5-methyl-Pyrazine | 17.21 | 1386 | 1387 | 121 | 122-94 | 98 | -65.21 | -18.77 | -18.27 | -26.70 |
| 58 | 2,3,5-Trimethyl-Pyrazine | 17.67 | 1397 | 1394 | 122 | 42-39-81 | 94 | -50.68 | -2.96 | -3.57 | -11.81 |
| 59 | 2-Ethyl-3-methylpyrazine* | 17.76 | 1400 | 1397 | 121 | 122-94-67 | 79 | -64.47 | -19.53 | -16.08 | -20.59 |
| 60 | 2-Methyl-3(2H)-furanone | 18.14 | 1409 | 1397 | 54 | 98-43-59 | 92 | -95.56 | -77.49 | -71.73 | -86.79 |
| 61 | 2-(n-propyl)-Pyrazine | 18.33 | 1413 | 1404 | 94 | 107-122 | 95 | -64.41 | -22.99 | -22.86 | -34.60 |
| 62 | 2,6-diethyl-Pyrazine | 19.01 | 1430 | 1415 | 135 | 136-175 | 96 | -44.72 | -23.19 | -23.37 | -19.24 |
| 63 | 2-Furfurylthiol* | 19.38 | 1439 | 1434 | 81 | 53-114 | 64 | -100.00 | -100.00 | -100.00 | -100.00 |
| 64 | 2-ethyl-3,5-dimethyl-Pyrazine | 19.43 | 1440 | 1435 | 135 | 136-42 | 95 | -38.72 | -28.82 | -21.70 | -18.98 |
| 65 | 2,3-Diethylpyrazine* | 19.86 | 1451 | 1458 | 135 | 136-56-108 | 85 | 81.92 | 89.61 | 115.20 | 93.88 |
| 66 | Acetic acid* | 20.18 | 1461 | 1465 | 60 | 43-45 | 84 | -44.82 | -22.89 | -20.52 | -17.03 |
| 67 | Furfural* | 20.52 | 1467 | 1467 | 96 | 95-39 | 87 | -68.79 | -38.45 | -35.34 | -64.13 |
| 68 | trans-Linalool oxide* | 20.62 | 1469 | 1472 | 59 | 94-55-93 | 93 | -20.50 | -31.54 | -43.72 | -36.60 |
| 69 | Acetoxyacetone | 20.79 | 1474 | 1470 | 43 | 86-116-73 | 98 | -56.87 | 4.86 | 7.71 | -43.39 |
| 70 | 2-Methyl-6-vinyl pyrazine | 21.25 | 1485 | 1488 | 120 | 52-121 | 98 | -73.05 | -68.67 | -66.57 | -76.99 |
| 71 | Furfuryl methyl sulfide* | 21.40 | 1488 | 1493 | 81 | 128-53 | 97 | -100.00 | 4.89 | -20.66 | -58.08 |
| 72 | 3,5-Diethyl-2-methyl-pyrazine | 21.46 | 1490 | 1496 | 149 | 150-120 | 96 | -19.69 | -30.11 | -19.18 | -21.63 |
| 73 | 2,5-Dimethyl-3(2H)-furanone* | 21.72 | 1496 | 1492 | 40 | 68-112 | 97 | -83.62 | -31.17 | -31.05 | -64.88 |
| 74 | 2,5-Hexanedione* | 21.93 | 1501 | 1505 | 43 | 99-114 | 96 | -20.82 | 9.34 | 2.59 | -48.78 |
| 75 | 2-Acetylfuran | 22.05 | 1505 | 1510 | 95 | 110-126 | 98 | -53.67 | 27.11 | 16.78 | -17.42 |
| 76 | 4-Vinyltetrahydro-2H-pyran-2-one | 22.73 | 1522 | - | 67 | 54-126 | 93 | -98.02 | -94.02 | -82.92 | -97.51 |
| 77 | 1-(2-Furyl)-2-propanone | 22.80 | 1523 | 1524 | 81 | 124-116 | 95 | -94.75 | 18.43 | -2.16 | -51.57 |
| 78 | 2,3-Dimethyl-2-cyclopenten-1-one* | 23.00 | 1528 | 1530 | 67 | 110-95 | 94 | -33.81 | -7.82 | -14.35 | -35.06 |
| 79 | 2-oxopropyl propanoate | 23.31 | 1536 | - | 57 | 43-100-87 | 98 | -47.94 | -3.10 | -9.32 | -41.40 |
| 80 | 1-Acetoxy-2-butanone | 23.49 | 1540 | 1536 | 57 | 43-100-87 | 97 | -43.87 | -1.30 | -7.75 | -41.96 |
| 81 | Furfuryl acetate* | 23.66 | 1545 | 1541 | 81 | 98-140 | 99 | -58.77 | 10.41 | -6.46 | -34.68 |
| 82 | Propanoic acid* | 23.11 | 1531 | 1555 | 74 | 45-57 | 92 | 164.69 | 103.41 | 94.90 | 112.32 |
| 83 | 5-Methyl-2-furancarboxaldehyde | 24.77 | 1573 | 1570 | 110 | 53-81 | 98 | -34.67 | -5.68 | -8.44 | -47.87 |
| 84 | 2-Propionylfuran* | 24.86 | 1575 | 1571 | 95 | 124-99 | 90 | -40.96 | 12.05 | -2.07 | -22.46 |
| 85 | (5-Methyl-2-furyl)methanethiol | 25.07 | 1580 | - | 95 | 138-96 | 98 | -93.81 | 9.07 | -9.72 | -58.88 |

| | | | | | | | | | | | |
|---|---|---|---|---|---|---|---|---|---|---|---|
| 86 | (1-methylethenyl)-Pyrazine | 25.41 | 1589 | - | 119 | 120-131 | 94 | -28.97 | -16.99 | -19.66 | -42.36 |
| 87 | 2-Acetylpyridine* | 25.72 | 1597 | 1602 | 78 | 134-105 | 95 | -47.45 | -0.70 | -10.46 | -32.11 |
| 88 | Furfuryl propanoate* | 26.00 | 1604 | 1606 | 81 | 98-154 | 85 | -23.44 | 18.52 | -11.95 | -27.68 |
| 89 | 5H-5-Methyl-6,7-dihydrocyclopentapyrazine* | 26.21 | 1609 | 1616 | 119 | 134-137 | 92 | -24.49 | -23.80 | -23.56 | -31.82 |
| 90 | 1-methyl 1H-Pyrrole-2-carboxaldehyde* | 26.46 | 1616 | 1620 | 109 | 108-53-80 | 92 | -36.14 | 4.63 | -1.09 | -25.06 |
| 91 | 4-hydroxy-butanoic acid | 26.66 | 1621 | - | 42 | 41-86 | 97 | 81.26 | 41.49 | 33.11 | 5.89 |
| 92 | 2-Isopropenylpyrazine | 27.01 | 1630 | - | 120 | 119-93-65 | 86 | -21.16 | -24.96 | -24.54 | -39.47 |
| 93 | Butanoic acid | 26.59 | 1619 | 1628 | 60 | 87-112 | 84 | nd | nd | nd | nd |
| 94 | 2,5-dihydro-3,5-dimethyl-2-Furanone | 27.48 | 1642 | 1640 | 69 | 112-97 | 85 | -9.31 | 9.75 | 7.66 | -27.89 |
| 95 | 1-(2-Furyl)-butan-3-one | 27.58 | 1645 | 1651 | 81 | 138-67 | 95 | -23.97 | 4.81 | -13.48 | -38.62 |
| 96 | 2-Acetyl-1-methylpyrrole* | 27.77 | 1650 | 1657 | 108 | 123-53 | 95 | -24.95 | -6.54 | -16.90 | -37.12 |
| 97 | 3-Mercapto-3-methyl-1-butanol | 28.17 | 1660 | 1658 | 41 | 69-71 | 97 | -100.00 | -43.63 | -61.47 | -94.05 |
| 98 | Furfuryl alcohol* | 28.49 | 1669 | 1660 | 98 | 81-69 | 99 | 30.78 | 25.40 | 20.50 | -20.48 |
| 99 | Unknown 3’ (m/z 126; 84; 97) | 28.74 | 1675 | - | 126 | 84-97 | 76 | -19.18 | -2.95 | -17.23 | -29.11 |
| 100 | 3-Methyl-butanoic acid | 29.74 | 1701 | 1680 | 60 | 43-87 | 92 | 15.30 | -1.38 | 3.60 | 8.94 |
| 101 | 2-Furfuryl-5-methylfuran | 28.44 | 1667 | 1678 | 162 | 91-119 | 79 | nd | nd | nd | nd |
| 102 | 2-Acetyl-3-methylpyrazine* | 29.08 | 1684 | 1686 | 136 | 94-67-108 | 77 | -14.34 | -12.87 | -17.26 | -37.22 |
| 103 | Unknown 4 (m/z 134; 133; 81) | 29.65 | 1699 | - | 134 | 133-81 | 95 | -15.09 | -23.75 | -22.06 | -39.76 |
| 104 | Unknown 5 (m/z 97; 69; 126) | 30.29 | 1716 | - | 97 | 69-126 | 83 | -7.16 | -3.12 | -8.66 | -32.18 |
| 105 | N-acetyl-4(H)-Pyridine | 30.45 | 1720 | - | 80 | 123-53 | 94 | -53.74 | -16.85 | -15.76 | -48.11 |
| 106 | 3-Methoxy-2-methyl-cyclohex-2-enone | 30.77 | 1729 | - | 140 | 111-97 | 83 | -69.30 | -13.64 | -22.16 | -42.05 |
| 107 | 3-ethyl-4-methyl-2,5-Furandione | 30.92 | 1733 | - | 67 | 53-140 | 94 | -17.65 | -13.30 | -30.17 | -55.12 |
| 108 | 2(5H)-Furanone* | 30.64 | 1726 | 1712 | 55 | 84-150 | 90 | nd | nd | nd | nd |
| 109 | Methyl salicylate* | 31.45 | 1748 | 1753 | 98 | 55-137 | 65 | nd | nd | nd | nd |
| 110 | Unknown 6 (m/z 95; 138; 150) | 32.46 | 1775 | - | 95 | 138-150 | 95 | -13.93 | -28.16 | -35.84 | -56.57 |
| 111 | Unknown 7 (m/z 94; 137; 78) | 32.63 | 1780 | - | 94 | 137-78 | 94 | -93.79 | -76.21 | -63.80 | -93.55 |
| 112 | 3,5-Dimethyl cyclopentenolone | 33.15 | 1794 | - | 126 | 69-111 | 91 | 4.00 | -6.84 | -13.06 | -23.88 |

| | | | | | | | | | | | |
|---|---|---|---|---|---|---|---|---|---|---|---|
| 113 | Unknown 8 (m/z 80; 137; 109) | 33.18 | 1795 | - | 80 | 137-109 | 89 | -33.53 | -14.18 | -18.38 | -48.51 |
| 114 | 3-methyl-2-Butenoic acid | 32.77 | 1784 | 1804 | 100 | 55-83 | 92 | nd | nd | nd | nd |
| 115 | Unknown 9 (m/z 55; 83; 126) | 33.84 | 1813 | - | 55 | 83-126 | 83 | 0.04 | -2.28 | -9.25 | -22.08 |
| 116 | 2-hydroxy-3-methyl-2-Cyclopenten-1-one | 34.27 | 1826 | 1807 | 112 | 69-83 | 94 | 28.86 | 11.02 | 3.04 | -14.58 |
| 117 | 1-(2-furanylmethyl)-1H-Pyrrole | 34.39 | 1829 | 1833 | 81 | 147-53 | 97 | -14.57 | -3.21 | -16.43 | -43.72 |
| 118 | Unknown 10 (m/z 128; 112; 151) | 35.23 | 1853 | - | 128 | 112-151 | 84 | -84.92 | 3.19 | -1.89 | -26.20 |
| 119 | n-butylbenzoate | 35.37 | 1857 | | 105 | 123-60 | 86 | 3.88 | -51.13 | -14.19 | -6.97 |
| 120 | 2-methoxy-Phenol | 35.44 | 1859 | 1859 | 109 | 124-81 | 98 | 6.00 | 5.19 | -14.36 | -37.37 |
| 121 | Unknown 11 (m/z 109; 53; 152) | 35.55 | 1862 | - | 109 | 53-152 | 97 | -9.38 | -19.46 | -22.41 | -54.18 |
| 122 | 3-ethyl-2-hydroxy- 2-Cyclopenten-1-one | 36.55 | 1890 | 1891 | 126 | 83-97 | 87 | 6.79 | -1.85 | -11.39 | -32.63 |
| 123 | Unknown 12 (m/z 81; 161; 53) | 36.80 | 1898 | - | 81 | 161-53 | 94 | -49.90 | -31.58 | -32.12 | -75.80 |
| 124 | Phenylethyl Alcohol* | 37.14 | 1908 | 1914 | 91 | 65-122 | 92 | 9.10 | -27.43 | -16.25 | -53.71 |
| 125 | 2-Thiophenemethanol* | 38.31 | 1942 | 1950 | 114 | 85-97 | 91 | 60.09 | 16.15 | 7.91 | -24.96 |
| 126 | Maltol* | 38.83 | 1958 | 1952 | 126 | 71-97 | 98 | 49.28 | 28.83 | -1.55 | 3.42 |
| 127 | 2-Acetylpyrrole* | 39.18 | 1968 | 1966 | 94 | 109-66 | 99 | 56.64 | 25.79 | 16.67 | -12.84 |
| 128 | 4(1H)-Quinazolinone | 39.51 | 1978 | - | 146 | 93-118 | 92 | -6.37 | -8.40 | -7.80 | -44.99 |
| 129 | Difurfuryl ether | 39.75 | 1985 | 1980 | 81 | 82-178 | 97 | 1.12 | 8.04 | -9.83 | -44.57 |
| 130 | 4-Hydroxy-3-methylacetophenone* | 40.10 | 1996 | - | 135 | 150-107 | 88 | -1.37 | -5.05 | -12.10 | -45.44 |
| 131 | Phenol* | 40.59 | 2010 | 1994 | 94 | 66-108 | 94 | 79.79 | 38.43 | 15.42 | -10.38 |
| 132 | 1H-Pyrrole-2-carboxaldehyde* | 40.90 | 2020 | 2012 | 95 | 66-39 | 99 | 133.27 | 59.47 | 46.09 | 20.86 |
| 133 | 4-ethyl guaiacol | 41.15 | 2028 | 2034 | 137 | 152-122 | 95 | -3.27 | -16.10 | -18.79 | -42.21 |
| 134 | 2,5-Dimethyl-4-hydroxy-3(2H)-furanone (Furaneol)* | 41.44 | 2037 | 2039 | 43 | 128-85 | 73 | -100.00 | -51.51 | -27.84 | -79.86 |
| 135 | 5-Acetyldihydro-2(3H)-furanone (Solerone) | 42.00 | 2054 | 2096 | 85 | 57-128 | 96 | 6.71 | -18.08 | -18.14 | -39.78 |
| 136 | N-Methyl-2-formylpyrrol | 43.47 | 2100 | - | 109 | 108-80 | 91 | 59.35 | 34.05 | 34.82 | -10.28 |
| 137 | Unknown 13 (m/z 57; 99; 149) | 44.24 | 2125 | - | 57 | 99-149 | 75 | -100.00 | -12.07 | -21.41 | -54.20 |
| 138 | 4-Vinyl guaiacol* | 46.35 | 2193 | 2185 | 150 | 135-107 | 99 | -52.34 | -71.37 | 89.29 | -16.57 |

| | | | | | | | | | | |
|---|---|---|---|---|---|---|---|---|---|---|
| 139 | Nonanoic acid* | 46.87 | 2210 | 2168 | 60 | 73-57-41 | 95 | -60.06 | -44.67 | -47.97 | -77.21 |
| 140 | Unknown 14 (m/z 81; 175; 163) | 47.64 | 2236 | - | 81 | 175-163 | 93 | -2.30 | 3.29 | -25.33 | -31.64 |
| 141 | n-Decanoic acid* | 50.10 | 2319 | 2303 | 60 | 57-129 | 92 | -7.90 | -49.05 | 70.72 | -38.97 |
| 142 | 2-Benzofuran-1(3H)-one | 50.39 | 2329 | 2356 | 105 | 77-134 | 90 | 20.23 | -53.08 | -30.52 | 2.59 |
| 143 | Unknown 15 (m/z 95; 39; 67) | 51.95 | 2384 | - | 95 | 39-67 | 95 | -69.61 | -40.42 | -55.80 | -75.81 |
| 144 | 2,3-dihydro-Benzofuran | 52.27 | 2395 | - | 120 | 91-119-65 | 84 | nd | nd | nd | nd |
| 145 | Indole* | 53.32 | 2434 | 2448 | 117 | 90-89-63 | 89 | -52.19 | -12.20 | -20.45 | -57.89 |
| 146 | Benzoic acid* | 53.27 | 2432 | 2433 | 105 | 122-77 | 84 | nd | nd | nd | nd |
| 147 | 5-(Hydroxymethyl)dihydro-2(3H)-furanone* | 54.22 | 2467 | - | 85 | 57-43 | 92 | 227.90 | 71.38 | 25.43 | 47.26 |
