# Supplementary material for: Potential Aroma Chemical Fingerprint of Oxidised Coffee Note by HS-SPME-GC-MS and Machine Learning
Source: Foods. 2022 Dec 16;11(24):4083. doi: 10.3390/foods11244083 (PMC9778272; doi:10.3390/foods11244083)
Supplement: Supplementary file 1 [file foods-11-04083-s001.zip › Figure S1.pptx]

## Slide 1
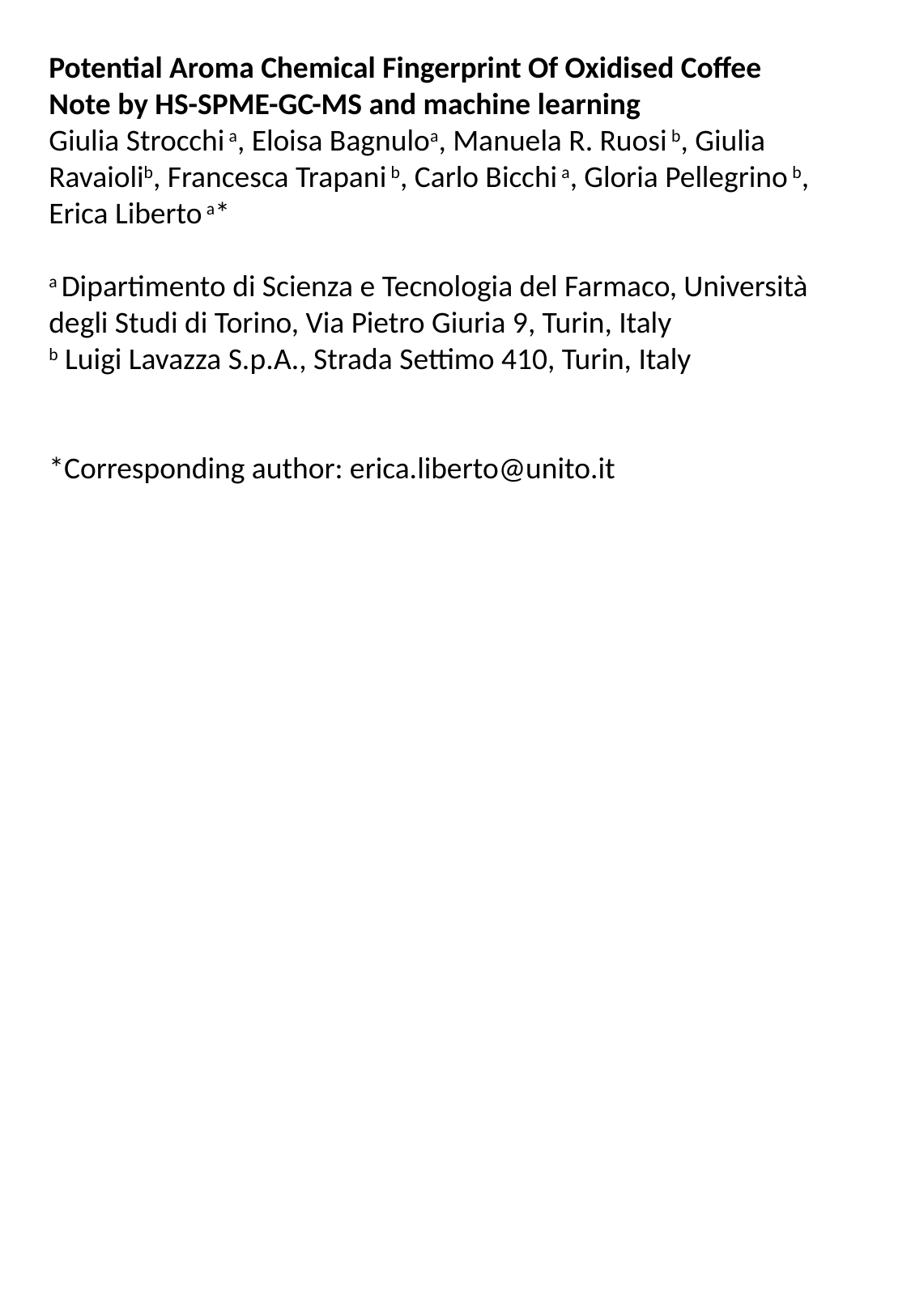

Potential Aroma Chemical Fingerprint Of Oxidised Coffee Note by HS-SPME-GC-MS and machine learning
Giulia Strocchi a, Eloisa Bagnuloa, Manuela R. Ruosi b, Giulia Ravaiolib, Francesca Trapani b, Carlo Bicchi a, Gloria Pellegrino b, Erica Liberto a*
a Dipartimento di Scienza e Tecnologia del Farmaco, Università degli Studi di Torino, Via Pietro Giuria 9, Turin, Italy
b Luigi Lavazza S.p.A., Strada Settimo 410, Turin, Italy
*Corresponding author: erica.liberto@unito.it

## Slide 2
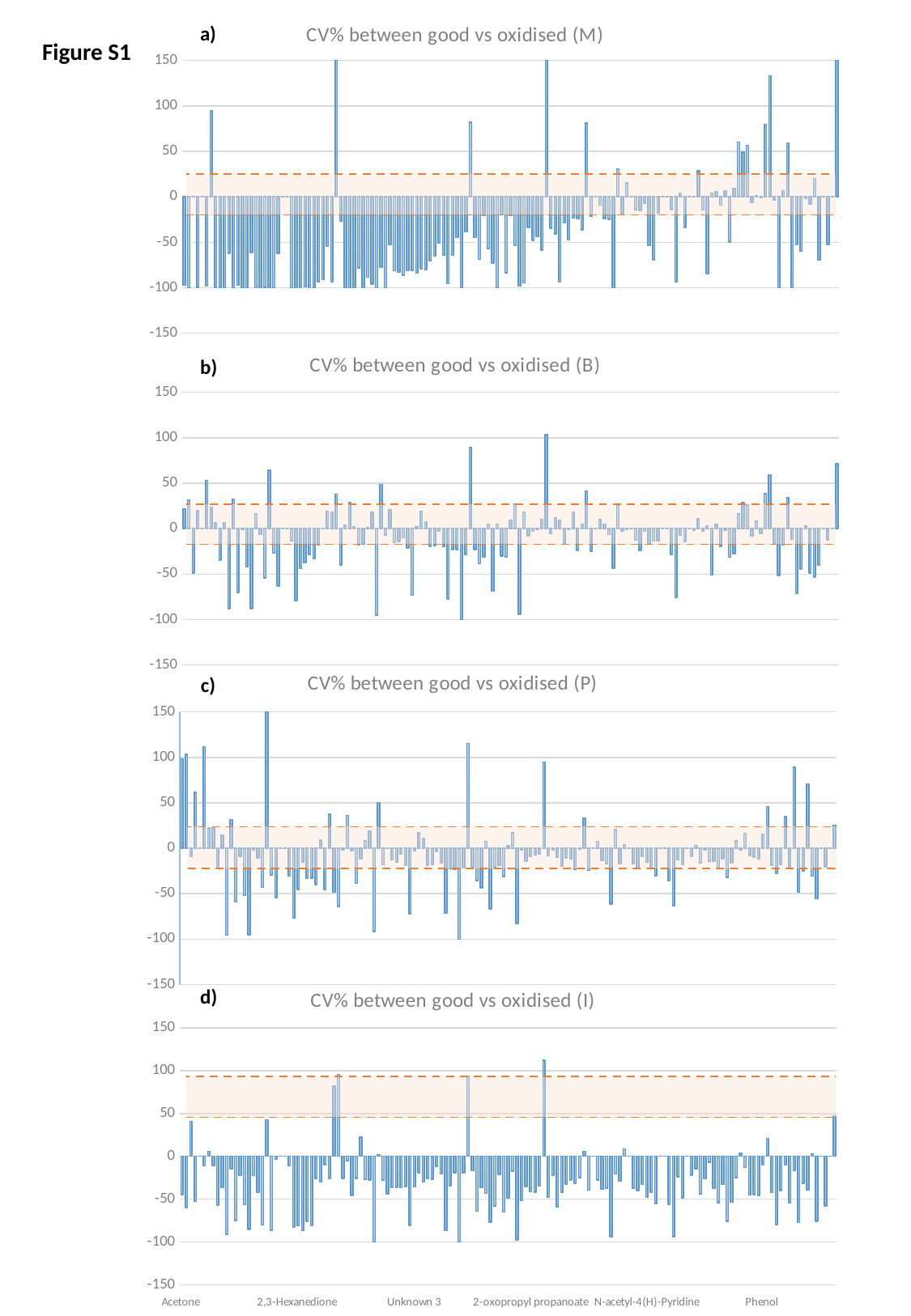

### Chart: CV% between good vs oxidised (M)
| Category | |
|---|---|
| Acetone | -97.26741660892421 |
| Methyl acetate | -100.0 |
| Tetrahydrofuran | 0.0 |
| 2-Methyl-furan | -99.54708391463683 |
| 2,4-Dimethyl-1-heptene | 0.0 |
| 2-Butanone | -98.09799794999434 |
| 2,3-Dihydro-5-methyl-Furane | 95.03982346877791 |
| 2-Methyl butanal | -99.57066405095357 |
| 3-Methyl butanal | -99.37153112680832 |
| 2,5-dimethyl-furane | -100.0 |
| 1-Methyl piperidine | -62.27425331429786 |
| Unknown 1 | -100.0 |
| 2,3-Butanedione | -97.2196931908251 |
| Methyl 3-methylbutanoate | -100.0 |
| Thiophene | -100.0 |
| N-Methyl-1,2,5,6-tetrahydropyridine | -61.2276094950304 |
| 3-Hexanone | -100.0 |
| Unknown 2 | -100.0 |
| 2,3-Pentanedione | -99.44085746824702 |
| Dimethyl disulfide | -100.0 |
| 4-Vinylfuran | -100.0 |
| Hexanal | -62.17212955654189 |
| Tridecanol | 0.0 |
| 3,3,5-Trimethyl-1,5-heptadiene | 0.0 |
| 4,5-Dimethyl-2-undecene | -100.0 |
| 3-penten-2-one | -100.0 |
| 2,3-Hexanedione | -100.0 |
| 1-Methyl-1H-pyrrole | -99.26607522998509 |
| 3,4-Hexandione | -100.0 |
| 2-Vinyl-5-methylfuran | -99.62257131157988 |
| beta-Myrcene | -93.48626801536066 |
| Pyridine | -91.20240176233392 |
| Limonene | -54.756626747752335 |
| Pyrazine | -93.84321148956845 |
| Butyl butanoate | 178.8521881489791 |
| 2-n-Pentylfuran | -27.125956525783195 |
| 2-Furfuryl methyl ether | -100.0 |
| Thiazole | -100.0 |
| 3-Methyl-3-buten-1-ol | -100.0 |
| E-beta Ocimene | -78.07022464495012 |
| 3-Methyl-2-butenyl acetate | -100.0 |
| Methyl-pyrazine | -88.20423795458593 |
| Dihydro-2-methyl-3(2H)-furanone | -96.01283839254128 |
| 2,5-Dimethyl-1H-pyrrole | -100.0 |
| 3-Hydroxy-2-butanone | -77.63873116251953 |
| trans-2-Methyl-5-n-propenylfuran | -100.0 |
| 1-Hydroxy-2-propanone | -52.63697995034662 |
| 2,5-Dimethyl-pyrazine | -81.37220634599721 |
| 2,6-Dimethyl-pyrazine | -83.13422450121757 |
| 2-Ethyl-pyrazine | -85.99975466663605 |
| 2,3-Dimethyl-Pyrazine+2-Hydroxyisobutyric acid | -81.22846941160243 |
| 2-Cyclopenten-1-one | -80.81643893024663 |
| Unknown 3 | -84.0969869964748 |
| 2-Hydroxy-3-pentanone | -79.27333830540071 |
| 2-Methyl-2-cyclopenten-1-one | -80.08848124258606 |
| 2-Ethyl-6-methyl-Pyrazine | -70.20962211045678 |
| 2-Ethyl-5-methyl-Pyrazine | -65.20709089084266 |
| 2,3,5-Trimethyl-Pyrazine | -50.68158467177698 |
| 2-Ethyl-3-methylpyrazine | -64.46522326113877 |
| 2-Methyl-3(2H)furanon | -95.55974712188028 |
| 2-(n-propyl)-Pyrazine | -64.40564050611256 |
| 2,6-diethyl-Pyrazine | -44.71503542635975 |
| 2-Furfurylthiol | -100.0 |
| 2-ethyl-3,5-dimethyl-Pyrazine | -38.717297937514104 |
| Acetic acid | 81.92388508301977 |
| 2,3-Diethylpyrazine | -44.82129393896351 |
| Furfural | -68.7874376479059 |
| trans-Linalool oxide | -20.50208354111168 |
| Acetoxyacetone | -56.86731002856684 |
| 2-Methyl-6-vinyl pyrazine | -73.05135427204766 |
| Furfuryl methyl sulfide | -100.0 |
| 3,5-Diethyl-2-methyl-pyrazine | -19.68542452899009 |
| 2,5-Dimethyl-3(2H)-furanone | -83.62158886262685 |
| 2,5-Hexanedione | -20.815588364785018 |
| 2-Acetylfuran | -53.667444577023495 |
| 4-Vinyltetrahydro-2H-pyran-2-one | -98.01826648831302 |
| 1-(2-Furyl)-2-propanone | -94.7528439949738 |
| 2,3-Dimethyl-2-cyclopenten-1-one | -33.81085816807193 |
| 2-oxopropyl propanoate | -47.93745773716225 |
| 1-Acetoxy-2-butanone | -43.86996801045525 |
| Furfuryl acetate | -58.76752598806411 |
| Propanoic acid | 164.68899899695484 |
| 5-Methyl-2-furancarboxaldehyde | -34.66595271640891 |
| 2-Propionylfuran | -40.95829032872867 |
| (5-Methyl-2-furyl)methanethiol | -93.81355423260804 |
| (1-methylethenyl)-Pyrazine | -28.972688187318035 |
| 2-Acetylpyridine | -47.44599722772609 |
| Furfuryl propanoate | -23.441205182666526 |
| 5H-5-Methyl-6,7-dihydrocyclopentapyrazine | -24.49226333655324 |
| 1-methyl 1H-Pyrrole-2-carboxaldehyde | -36.13929056371546 |
| 4-hydroxy-butanoic acid | 81.26026130904656 |
| 2-Isopropenylpyrazine | -21.15956647776677 |
| Butanoic acid | 0.0 |
| 2,5-dihydro-3,5-dimethyl-2-Furanone | -9.306019802363771 |
| 1-(2-Furyl)-butan-3-one | -23.966560530977986 |
| 2-Acetyl-1-methylpyrrole | -24.953384404356406 |
| 3-Mercapto-3-methyl-1-butanol | -100.0 |
| Furfuryl alcohol | 30.775178233840673 |
| 3-Ethyl-2-hydroxy-2-cyclopenten-1-one | -19.18199270207439 |
| 3-Methyl-butanoic acid | 15.300402124603071 |
| 2-Furfuryl-5-methylfuran | 0.0 |
| Acetyl-6-methylpyrazine | -14.336542636332444 |
| Unknown 4 | -15.08862959237281 |
| Unknown 5 | -7.161226406924665 |
| N-acetyl-4(H)-Pyridine | -53.73866373452061 |
| 3-Methoxy-2-methyl-cyclohex-2-enone | -69.296821587164 |
| 3-ethyl-4-methyl-2,5-Furandione | -17.652311658366973 |
| 2(5H)-Furanone | 0.0 |
| Methyl salicylate | 0.0 |
| Unknown 6 | -13.926868933853626 |
| Unknown 7 | -93.7899726670097 |
| 3,5-DIMETHYL CYCLOPENTENOLONE | 4.000050476260273 |
| Unknown 8 | -33.534458737766556 |
| 3-methyl-2-Butenoic acid | 0.0 |
| Unknown 9 | 0.0366936772503451 |
| 2-hydroxy-3-methyl-2-Cyclopenten-1-one | 28.85668613020981 |
| 1-(2-furanylmethyl)-1H-Pyrrole | -14.568117510019531 |
| Unknown 10 | -84.91925884266756 |
| n-butylbenzoate | 3.8756685889794196 |
| 2-methoxy-Phenol | 6.004479842730327 |
| Unknown 11 | -9.382889465943313 |
| 3-ethyl-2-hydroxy- 2-Cyclopenten-1-one | 6.7919396052627246 |
| Unknown 12 | -49.8993944501564 |
| Phenylethyl Alcohol | 9.103723989547271 |
| 2-Thiophenemethanol | 60.086352302252756 |
| Maltol | 49.27660445160166 |
| 2-Acetylpyrrole | 56.639477827443606 |
| 4(1H)-Quinazolinone | -6.372815094007834 |
| Difurfuryl ether | 1.1240119192696674 |
| 4-Hydroxy-3-methylacetophenone | -1.3689254261064023 |
| Phenol | 79.78687985562783 |
| 1H-Pyrrole-2-carboxaldehyde | 133.27186316653078 |
| 4-ethyl guaiacol | -3.2670701606763073 |
| 2,5-Dimethyl-4-hydroxy-3(2H)-furanone (Furaneol) | -100.0 |
| 5-Acetyldihydro-2(3H)-furanone (Solerone) | 6.712818341131039 |
| N-Methyl-2-formylpyrrol | 59.352197244223284 |
| Unknown 13 | -100.0 |
| Nonanoic acid | -52.33859746305758 |
| 2-Vinyl guaiacol | -60.05863796614504 |
| Unknown 14 | -2.3041577801082234 |
| n-Decanoic acid | -7.899483375281491 |
| 2-Benzofuran-1(3H)-one | 20.234766338471797 |
| 2,3-dihydro-Benzofuran | -69.61222327456768 |
| Unknown 15 | 0.0 |
| Indole | -52.19332749732517 |
| Benzoic acid | 0.0 |
| 5-(Hydroxymethyl)dihydro-2(3H)-furanone | 227.89997254348262 |
### Chart: CV% between good vs oxidised (B)
| Category | |
|---|---|
| Acetone | 21.5010461810413 |
| Methyl acetate | 31.44524691226682 |
| Tetrahydrofuran | -49.01345306857761 |
| 2-Methyl-furan | 19.612273245520345 |
| 2,4-Dimethyl-1-heptene | 0.0 |
| 2-Butanone | 53.26089201658847 |
| 2,3-Dihydro-5-methyl-Furane | 23.619952207875645 |
| 2-Methyl butanal | 6.248693988729165 |
| 3-Methyl butanal | -34.47618661935293 |
| 2,5-dimethyl-furane | 6.619266646275412 |
| 1-Methyl piperidine | -88.10446165276262 |
| Unknown 1 | 32.649148626753956 |
| 2,3-Butanedione | -70.2016305597437 |
| Methyl 3-methylbutanoate | -0.8485149164982423 |
| Thiophene | -41.93620723193636 |
| N-Methyl-1,2,5,6-tetrahydropyridine | -87.96827034654586 |
| 3-Hexanone | 16.551222071150992 |
| Unknown 2 | -6.6625003505313956 |
| 2,3-Pentanedione | -54.21338116266313 |
| Dimethyl disulfide | 64.47627234712165 |
| 4-Vinylfuran | -27.20539855683221 |
| Hexanal | -63.17099999330647 |
| Tridecanol | 0.0 |
| 3,3,5-Trimethyl-1,5-heptadiene | 0.0 |
| 4,5-Dimethyl-2-undecene | -13.397344114096407 |
| 3-penten-2-one | -79.3120021256532 |
| 2,3-Hexanedione | -44.12049643719867 |
| 1-Methyl-1H-pyrrole | -37.86204370461434 |
| 3,4-Hexandione | -28.390857320893836 |
| 2-Vinyl-5-methylfuran | -32.72225902809257 |
| beta-Myrcene | -17.86098897122985 |
| Pyridine | 0.29219583907000285 |
| Limonene | 19.099113627249636 |
| Pyrazine | 18.49472336090024 |
| Butyl butanoate | 38.131113676408056 |
| 2-n-Pentylfuran | -39.78568810183018 |
| 2-Furfuryl methyl ether | 4.015956723478235 |
| Thiazole | 28.638076041556143 |
| 3-Methyl-3-buten-1-ol | 2.2729033080669327 |
| E-beta Ocimene | -18.0755417732921 |
| 3-Methyl-2-butenyl acetate | -16.603903284616994 |
| Methyl-pyrazine | 1.1969460116263664 |
| Dihydro-2-methyl-3(2H)-furanone | 18.003898391786276 |
| 2,5-Dimethyl-1H-pyrrole | -95.09364383343853 |
| 3-Hydroxy-2-butanone | 48.60603988515049 |
| trans-2-Methyl-5-n-propenylfuran | -6.92687834644607 |
| 1-Hydroxy-2-propanone | 21.221054689197043 |
| 2,5-Dimethyl-pyrazine | -15.170283428998516 |
| 2,6-Dimethyl-pyrazine | -14.269930061420435 |
| 2-Ethyl-pyrazine | -10.168798234248491 |
| 2,3-Dimethyl-Pyrazine+2-Hydroxyisobutyric acid | -21.0507483949328 |
| 2-Cyclopenten-1-one | -73.26520404014467 |
| Unknown 3 | 2.4402243294923074 |
| 2-Hydroxy-3-pentanone | 19.00634873908765 |
| 2-Methyl-2-cyclopenten-1-one | 7.494658504381232 |
| 2-Ethyl-6-methyl-Pyrazine | -19.380328871788564 |
| 2-Ethyl-5-methyl-Pyrazine | -18.76822975543468 |
| 2,3,5-Trimethyl-Pyrazine | -2.9619300532723183 |
| 2-Ethyl-3-methylpyrazine | -19.532580610896304 |
| 2-Methyl-3(2H)furanon | -77.48982300225161 |
| 2-(n-propyl)-Pyrazine | -22.987062408004874 |
| 2,6-diethyl-Pyrazine | -23.188984712665174 |
| 2-Furfurylthiol | -100.0 |
| 2-ethyl-3,5-dimethyl-Pyrazine | -28.818788587060357 |
| Acetic acid | 89.60898738690425 |
| 2,3-Diethylpyrazine | -22.8871264618755 |
| Furfural | -38.45081863916097 |
| trans-Linalool oxide | -31.535996171057434 |
| Acetoxyacetone | 4.86486737393015 |
| 2-Methyl-6-vinyl pyrazine | -68.66739496832429 |
| Furfuryl methyl sulfide | 4.886176216495198 |
| 3,5-Diethyl-2-methyl-pyrazine | -30.10601854468708 |
| 2,5-Dimethyl-3(2H)-furanone | -31.166369546044887 |
| 2,5-Hexanedione | 9.33625553546443 |
| 2-Acetylfuran | 27.11222560167362 |
| 4-Vinyltetrahydro-2H-pyran-2-one | -94.01939752303005 |
| 1-(2-Furyl)-2-propanone | 18.426687649597966 |
| 2,3-Dimethyl-2-cyclopenten-1-one | -7.818578335384374 |
| 2-oxopropyl propanoate | -3.1023144387776993 |
| 1-Acetoxy-2-butanone | -1.2952933482776934 |
| Furfuryl acetate | 10.407926238319568 |
| Propanoic acid | 103.40688910686117 |
| 5-Methyl-2-furancarboxaldehyde | -5.683511633129718 |
| 2-Propionylfuran | 12.04807029978929 |
| (5-Methyl-2-furyl)methanethiol | 9.067192607852071 |
| (1-methylethenyl)-Pyrazine | -16.990849263975065 |
| 2-Acetylpyridine | -0.6991031513123651 |
| Furfuryl propanoate | 18.522840474505326 |
| 5H-5-Methyl-6,7-dihydrocyclopentapyrazine | -23.7995941831301 |
| 1-methyl 1H-Pyrrole-2-carboxaldehyde | 4.6325732648320095 |
| 4-hydroxy-butanoic acid | 41.487205218038675 |
| 2-Isopropenylpyrazine | -24.96109878419873 |
| Butanoic acid | 0.0 |
| 2,5-dihydro-3,5-dimethyl-2-Furanone | 9.754200532575739 |
| 1-(2-Furyl)-butan-3-one | 4.810030668548981 |
| 2-Acetyl-1-methylpyrrole | -6.544562585224149 |
| 3-Mercapto-3-methyl-1-butanol | -43.62900859046934 |
| Furfuryl alcohol | 25.401213406119048 |
| 3-Ethyl-2-hydroxy-2-cyclopenten-1-one | -2.952407339928497 |
| 3-Methyl-butanoic acid | -1.38429520496758 |
| 2-Furfuryl-5-methylfuran | 0.0 |
| Acetyl-6-methylpyrazine | -12.865620560288843 |
| Unknown 4 | -23.74856633124324 |
| Unknown 5 | -3.1203376247284487 |
| N-acetyl-4(H)-Pyridine | -16.853958111466106 |
| 3-Methoxy-2-methyl-cyclohex-2-enone | -13.637039026696621 |
| 3-ethyl-4-methyl-2,5-Furandione | -13.298852385156101 |
| 2(5H)-Furanone | 0.0 |
| Methyl salicylate | 0.0 |
| Unknown 6 | -28.16354308658337 |
| Unknown 7 | -76.20537202701983 |
| 3,5-DIMETHYL CYCLOPENTENOLONE | -6.840352391874227 |
| Unknown 8 | -14.181232298241708 |
| 3-methyl-2-Butenoic acid | 0.0 |
| Unknown 9 | -2.281862169014763 |
| 2-hydroxy-3-methyl-2-Cyclopenten-1-one | 11.01887119226581 |
| 1-(2-furanylmethyl)-1H-Pyrrole | -3.2057841249885874 |
| Unknown 10 | 3.1899246184960353 |
| n-butylbenzoate | -51.13365293090758 |
| 2-methoxy-Phenol | 5.190353034940403 |
| Unknown 11 | -19.463063589137025 |
| 3-ethyl-2-hydroxy- 2-Cyclopenten-1-one | -1.8486480332002022 |
| Unknown 12 | -31.578279743461824 |
| Phenylethyl Alcohol | -27.43323762820935 |
| 2-Thiophenemethanol | 16.147966677154006 |
| Maltol | 28.83261681531055 |
| 2-Acetylpyrrole | 25.78849272585001 |
| 4(1H)-Quinazolinone | -8.398196107921304 |
| Difurfuryl ether | 8.042095443593018 |
| 4-Hydroxy-3-methylacetophenone | -5.045795308605898 |
| Phenol | 38.425061009972445 |
| 1H-Pyrrole-2-carboxaldehyde | 59.468191929774314 |
| 4-ethyl guaiacol | -16.101870590652187 |
| 2,5-Dimethyl-4-hydroxy-3(2H)-furanone (Furaneol) | -51.506764820379736 |
| 5-Acetyldihydro-2(3H)-furanone (Solerone) | -18.08180681547759 |
| N-Methyl-2-formylpyrrol | 34.05236927030483 |
| Unknown 13 | -12.072143413022406 |
| Nonanoic acid | -71.37294913987564 |
| 2-Vinyl guaiacol | -44.66750741928575 |
| Unknown 14 | 3.2936204460585405 |
| n-Decanoic acid | -49.045110796531866 |
| 2-Benzofuran-1(3H)-one | -53.082583270398196 |
| 2,3-dihydro-Benzofuran | -40.42127029741699 |
| Unknown 15 | 0.0 |
| Indole | -12.200316987756764 |
| Benzoic acid | 0.0 |
| 5-(Hydroxymethyl)dihydro-2(3H)-furanone | 71.37857686779444 |
### Chart: CV% between good vs oxidised (P)
| Category | Acetone |
|---|---|
| Acetone | 98.39189914953602 |
| Methyl acetate | 103.4047904230632 |
| Tetrahydrofuran | -9.194173142273248 |
| 2-Methyl-furan | 61.6019060067339 |
| 2,4-Dimethyl-1-heptene | 0.0 |
| 2-Butanone | 111.98350024709292 |
| 2,3-Dihydro-5-methyl-Furane | 22.36229508196721 |
| 2-Methyl butanal | 22.487220755883158 |
| 3-Methyl butanal | -21.753835968205067 |
| 2,5-dimethyl-furane | 14.550609903287365 |
| 1-Methyl piperidine | -95.87456598182601 |
| Unknown 1 | 31.858163826278197 |
| 2,3-Butanedione | -59.221730719662155 |
| Methyl 3-methylbutanoate | -8.82116306761496 |
| Thiophene | -52.005415709119404 |
| N-Methyl-1,2,5,6-tetrahydropyridine | -95.1685604368256 |
| 3-Hexanone | -2.183033633198766 |
| Unknown 2 | -10.895788202455751 |
| 2,3-Pentanedione | -43.10507684380868 |
| Dimethyl disulfide | 171.93577521324636 |
| 4-Vinylfuran | -29.466858789625366 |
| Hexanal | -54.60889916535078 |
| Tridecanol | 0.0 |
| 3,3,5-Trimethyl-1,5-heptadiene | 0.0 |
| 4,5-Dimethyl-2-undecene | -30.635608241896016 |
| 3-penten-2-one | -77.15372689588442 |
| 2,3-Hexanedione | -45.315261582947244 |
| 1-Methyl-1H-pyrrole | -15.681830074116975 |
| 3,4-Hexandione | -33.4494264600705 |
| 2-Vinyl-5-methylfuran | -33.07287170323972 |
| beta-Myrcene | -39.94243379525764 |
| Pyridine | 9.052549202916877 |
| Limonene | -45.30089404970724 |
| Pyrazine | 37.48932248273674 |
| Butyl butanoate | -48.61479636174033 |
| 2-n-Pentylfuran | -63.84679305302322 |
| 2-Furfuryl methyl ether | -1.6356474697757983 |
| Thiazole | 36.271840715156415 |
| 3-Methyl-3-buten-1-ol | -2.8313074701247216 |
| E-beta Ocimene | -38.52026245768497 |
| 3-Methyl-2-butenyl acetate | -11.959784408541854 |
| Methyl-pyrazine | 8.176035722064373 |
| Dihydro-2-methyl-3(2H)-furanone | 19.12489808449648 |
| 2,5-Dimethyl-1H-pyrrole | -91.86249280687676 |
| 3-Hydroxy-2-butanone | 50.30148674861166 |
| trans-2-Methyl-5-n-propenylfuran | -18.271727144060364 |
| 1-Hydroxy-2-propanone | 0.13689388510749487 |
| 2,5-Dimethyl-pyrazine | -12.901033725770644 |
| 2,6-Dimethyl-pyrazine | -14.930015694979435 |
| 2-Ethyl-pyrazine | -6.474637813280033 |
| 2,3-Dimethyl-Pyrazine+2-Hydroxyisobutyric acid | -19.289449736244986 |
| 2-Cyclopenten-1-one | -72.19386197504863 |
| Unknown 3 | -3.1524134108593658 |
| 2-Hydroxy-3-pentanone | 17.09951036564803 |
| 2-Methyl-2-cyclopenten-1-one | 10.574204964619664 |
| 2-Ethyl-6-methyl-Pyrazine | -18.960060177941312 |
| 2-Ethyl-5-methyl-Pyrazine | -18.267505085774552 |
| 2,3,5-Trimethyl-Pyrazine | -3.565934632972824 |
| 2-Ethyl-3-methylpyrazine | -16.075131271161244 |
| 2-Methyl-3(2H)furanon | -71.72568583441962 |
| 2-(n-propyl)-Pyrazine | -22.85581555150749 |
| 2,6-diethyl-Pyrazine | -23.365165991601014 |
| 2-Furfurylthiol | -100.0 |
| 2-ethyl-3,5-dimethyl-Pyrazine | -21.69946991273843 |
| Acetic acid | 115.19561062600478 |
| 2,3-Diethylpyrazine | -20.51584447960114 |
| Furfural | -35.335054267045834 |
| trans-Linalool oxide | -43.71582254491893 |
| Acetoxyacetone | 7.705205562590608 |
| 2-Methyl-6-vinyl pyrazine | -66.56642274550863 |
| Furfuryl methyl sulfide | -20.65872648149389 |
| 3,5-Diethyl-2-methyl-pyrazine | -19.181094125384725 |
| 2,5-Dimethyl-3(2H)-furanone | -31.053183038678426 |
| 2,5-Hexanedione | 2.589848423367086 |
| 2-Acetylfuran | 16.784415484677425 |
| 4-Vinyltetrahydro-2H-pyran-2-one | -82.92304150470352 |
| 1-(2-Furyl)-2-propanone | -2.164404770110637 |
| 2,3-Dimethyl-2-cyclopenten-1-one | -14.352809471168996 |
| 2-oxopropyl propanoate | -9.32471682337262 |
| 1-Acetoxy-2-butanone | -7.7493874667077565 |
| Furfuryl acetate | -6.458062728822646 |
| Propanoic acid | 94.90118051177544 |
| 5-Methyl-2-furancarboxaldehyde | -8.435451205089759 |
| 2-Propionylfuran | -2.073603381216757 |
| (5-Methyl-2-furyl)methanethiol | -9.718597212440528 |
| (1-methylethenyl)-Pyrazine | -19.663222165713762 |
| 2-Acetylpyridine | -10.464958841478357 |
| Furfuryl propanoate | -11.952341026433078 |
| 5H-5-Methyl-6,7-dihydrocyclopentapyrazine | -23.557173103619853 |
| 1-methyl 1H-Pyrrole-2-carboxaldehyde | -1.0949222639760052 |
| 4-hydroxy-butanoic acid | 33.11131441304179 |
| 2-Isopropenylpyrazine | -24.543690594623495 |
| Butanoic acid | 0.0 |
| 2,5-dihydro-3,5-dimethyl-2-Furanone | 7.6585866404267 |
| 1-(2-Furyl)-butan-3-one | -13.480091088238208 |
| 2-Acetyl-1-methylpyrrole | -16.896833898124783 |
| 3-Mercapto-3-methyl-1-butanol | -61.47463686999234 |
| Furfuryl alcohol | 20.495161217608317 |
| 3-Ethyl-2-hydroxy-2-cyclopenten-1-one | -17.23340606126663 |
| 3-Methyl-butanoic acid | 3.6007130414236954 |
| 2-Furfuryl-5-methylfuran | 0.0 |
| Acetyl-6-methylpyrazine | -17.256018442250205 |
| Unknown 4 | -22.059707559178086 |
| Unknown 5 | -8.657726080203018 |
| N-acetyl-4(H)-Pyridine | -15.763450551711061 |
| 3-Methoxy-2-methyl-cyclohex-2-enone | -22.155013305149904 |
| 3-ethyl-4-methyl-2,5-Furandione | -30.17020414407932 |
| 2(5H)-Furanone | 0.0 |
| Methyl salicylate | 0.0 |
| Unknown 6 | -35.840510730879416 |
| Unknown 7 | -63.80291174647326 |
| 3,5-DIMETHYL CYCLOPENTENOLONE | -13.056331739355889 |
| Unknown 8 | -18.381232484042496 |
| 3-methyl-2-Butenoic acid | 0.0 |
| Unknown 9 | -9.25159798591671 |
| 2-hydroxy-3-methyl-2-Cyclopenten-1-one | 3.044505458337815 |
| 1-(2-furanylmethyl)-1H-Pyrrole | -16.434253043704608 |
| Unknown 10 | -1.8880561073986983 |
| n-butylbenzoate | -14.190847763862205 |
| 2-methoxy-Phenol | -14.358377586409931 |
| Unknown 11 | -22.413300209462147 |
| 3-ethyl-2-hydroxy- 2-Cyclopenten-1-one | -11.38504230047789 |
| Unknown 12 | -32.12443674202879 |
| Phenylethyl Alcohol | -16.251431340150788 |
| 2-Thiophenemethanol | 7.9109391726572 |
| Maltol | -1.553301100500221 |
| 2-Acetylpyrrole | 16.672911448073492 |
| 4(1H)-Quinazolinone | -7.800088572176252 |
| Difurfuryl ether | -9.831275560917922 |
| 4-Hydroxy-3-methylacetophenone | -12.09541726926885 |
| Phenol | 15.418545622269821 |
| 1H-Pyrrole-2-carboxaldehyde | 46.08773760845799 |
| 4-ethyl guaiacol | -18.786334768034056 |
| 2,5-Dimethyl-4-hydroxy-3(2H)-furanone (Furaneol) | -27.837931817679323 |
| 5-Acetyldihydro-2(3H)-furanone (Solerone) | -18.13630625404271 |
| N-Methyl-2-formylpyrrol | 34.81935009292453 |
| Unknown 13 | -21.411421654193678 |
| Nonanoic acid | 89.2880880346967 |
| 2-Vinyl guaiacol | -47.966496378209676 |
| Unknown 14 | -25.327995789444874 |
| n-Decanoic acid | 70.7237248705361 |
| 2-Benzofuran-1(3H)-one | -30.51532936102382 |
| 2,3-dihydro-Benzofuran | -55.803287710723396 |
| Unknown 15 | 0.0 |
| Indole | -20.450032206248284 |
| Benzoic acid | 0.0 |
| 5-(Hydroxymethyl)dihydro-2(3H)-furanone | 25.429556804509424 |
### Chart: CV% between good vs oxidised (I)
| Category | |
|---|---|
| Acetone | -44.68927905923082 |
| Methyl acetate | -59.66569860167616 |
| Tetrahydrofuran | 40.88680911892221 |
| 2-Methyl-furan | -52.877433975346854 |
| 2,4-Dimethyl-1-heptene | 0.0 |
| 2-Butanone | -11.231656463189609 |
| 2,3-Dihydro-5-methyl-Furane | 5.749799856739588 |
| 2-Methyl butanal | -11.140148475390369 |
| 3-Methyl butanal | -57.250139103327044 |
| 2,5-dimethyl-furane | -36.44455968979288 |
| 1-Methyl piperidine | -90.85676974113048 |
| Unknown 1 | -15.128954249817342 |
| 2,3-Butanedione | -74.92727361386241 |
| Methyl 3-methylbutanoate | -22.555395228400318 |
| Thiophene | -56.382019029463336 |
| N-Methyl-1,2,5,6-tetrahydropyridine | -85.10553896140054 |
| 3-Hexanone | -22.279624268058875 |
| Unknown 2 | -42.108671019262935 |
| 2,3-Pentanedione | -79.91715081209922 |
| Dimethyl disulfide | 42.79627145429823 |
| 4-Vinylfuran | -86.08089454185111 |
| Hexanal | -3.674751325364436 |
| Tridecanol | 0.0 |
| 3,3,5-Trimethyl-1,5-heptadiene | 0.0 |
| 4,5-Dimethyl-2-undecene | -10.969935228988186 |
| 3-penten-2-one | -82.32461759089999 |
| 2,3-Hexanedione | -81.16357472543467 |
| 1-Methyl-1H-pyrrole | -86.76996261711906 |
| 3,4-Hexandione | -75.83808885839584 |
| 2-Vinyl-5-methylfuran | -81.11255236318901 |
| beta-Myrcene | -25.971801137910106 |
| Pyridine | -29.825279828819717 |
| Limonene | -9.572557873365925 |
| Pyrazine | -25.816575455837302 |
| Butyl butanoate | 82.24081435771194 |
| 2-n-Pentylfuran | 95.69208225628529 |
| 2-Furfuryl methyl ether | -25.914714204366085 |
| Thiazole | -4.721370210339665 |
| 3-Methyl-3-buten-1-ol | -45.64527691767971 |
| E-beta Ocimene | -25.829225036687813 |
| 3-Methyl-2-butenyl acetate | 22.331518389151448 |
| Methyl-pyrazine | -26.968508971205967 |
| Dihydro-2-methyl-3(2H)-furanone | -27.58771238888263 |
| 2,5-Dimethyl-1H-pyrrole | -100.0 |
| 3-Hydroxy-2-butanone | 1.9901326172512759 |
| trans-2-Methyl-5-n-propenylfuran | -27.639222658377527 |
| 1-Hydroxy-2-propanone | -43.67175947998197 |
| 2,5-Dimethyl-pyrazine | -36.52747437146133 |
| 2,6-Dimethyl-pyrazine | -35.93436017492323 |
| 2-Ethyl-pyrazine | -36.84909471402337 |
| 2,3-Dimethyl-Pyrazine+2-Hydroxyisobutyric acid | -35.152079391720605 |
| 2-Cyclopenten-1-one | -80.48371375332802 |
| Unknown 3 | -35.78301963593615 |
| 2-Hydroxy-3-pentanone | -19.20737909876759 |
| 2-Methyl-2-cyclopenten-1-one | -29.85995982740025 |
| 2-Ethyl-6-methyl-Pyrazine | -26.227669498355656 |
| 2-Ethyl-5-methyl-Pyrazine | -26.700423223876456 |
| 2,3,5-Trimethyl-Pyrazine | -11.808645905285085 |
| 2-Ethyl-3-methylpyrazine | -20.588756306548994 |
| 2-Methyl-3(2H)furanon | -86.79016730723478 |
| 2-(n-propyl)-Pyrazine | -34.60070771536394 |
| 2,6-diethyl-Pyrazine | -19.235893103721647 |
| 2-Furfurylthiol | -100.0 |
| 2-ethyl-3,5-dimethyl-Pyrazine | -18.97602644280705 |
| Acetic acid | 93.87739390200012 |
| 2,3-Diethylpyrazine | -17.03025363187419 |
| Furfural | -64.12523467992091 |
| trans-Linalool oxide | -36.598031453509975 |
| Acetoxyacetone | -43.389939726763174 |
| 2-Methyl-6-vinyl pyrazine | -76.99075679060752 |
| Furfuryl methyl sulfide | -58.076220558950766 |
| 3,5-Diethyl-2-methyl-pyrazine | -21.62908086659868 |
| 2,5-Dimethyl-3(2H)-furanone | -64.87632218372053 |
| 2,5-Hexanedione | -48.7771989769035 |
| 2-Acetylfuran | -17.417408200510426 |
| 4-Vinyltetrahydro-2H-pyran-2-one | -97.50917848864773 |
| 1-(2-Furyl)-2-propanone | -51.56516079025651 |
| 2,3-Dimethyl-2-cyclopenten-1-one | -35.05919721483956 |
| 2-oxopropyl propanoate | -41.39875379903654 |
| 1-Acetoxy-2-butanone | -41.9643211063283 |
| Furfuryl acetate | -34.68009959044767 |
| Propanoic acid | 112.3189450705352 |
| 5-Methyl-2-furancarboxaldehyde | -47.87273440705094 |
| 2-Propionylfuran | -22.460919476093487 |
| (5-Methyl-2-furyl)methanethiol | -58.883532499845835 |
| (1-methylethenyl)-Pyrazine | -42.35834420052677 |
| 2-Acetylpyridine | -32.11014489846968 |
| Furfuryl propanoate | -27.675754426167607 |
| 5H-5-Methyl-6,7-dihydrocyclopentapyrazine | -31.821904170972903 |
| 1-methyl 1H-Pyrrole-2-carboxaldehyde | -25.05606199068814 |
| 4-hydroxy-butanoic acid | 5.893943334263783 |
| 2-Isopropenylpyrazine | -39.468478275906484 |
| Butanoic acid | 0.0 |
| 2,5-dihydro-3,5-dimethyl-2-Furanone | -27.885902226002912 |
| 1-(2-Furyl)-butan-3-one | -38.617034389251806 |
| 2-Acetyl-1-methylpyrrole | -37.12151765261902 |
| 3-Mercapto-3-methyl-1-butanol | -94.04688988922257 |
| Furfuryl alcohol | -20.4807461388666 |
| 3-Ethyl-2-hydroxy-2-cyclopenten-1-one | -29.10786125012289 |
| 3-Methyl-butanoic acid | 8.944000294502095 |
| 2-Furfuryl-5-methylfuran | 0.0 |
| Acetyl-6-methylpyrazine | -37.221425647318476 |
| Unknown 4 | -39.75597405989106 |
| Unknown 5 | -32.17624984490691 |
| N-acetyl-4(H)-Pyridine | -48.10786075164713 |
| 3-Methoxy-2-methyl-cyclohex-2-enone | -42.05237491369319 |
| 3-ethyl-4-methyl-2,5-Furandione | -55.11802877884099 |
| 2(5H)-Furanone | 0.0 |
| Methyl salicylate | 0.0 |
| Unknown 6 | -56.57381132001673 |
| Unknown 7 | -93.55310538206881 |
| 3,5-DIMETHYL CYCLOPENTENOLONE | -23.879409597695247 |
| Unknown 8 | -48.511713424506524 |
| 3-methyl-2-Butenoic acid | 0.0 |
| Unknown 9 | -22.082383260006605 |
| 2-hydroxy-3-methyl-2-Cyclopenten-1-one | -14.577117662914857 |
| 1-(2-furanylmethyl)-1H-Pyrrole | -43.71725263771285 |
| Unknown 10 | -26.201367404375986 |
| n-butylbenzoate | -6.965182006852231 |
| 2-methoxy-Phenol | -37.37476311508685 |
| Unknown 11 | -54.18315555392642 |
| 3-ethyl-2-hydroxy- 2-Cyclopenten-1-one | -32.63296855539263 |
| Unknown 12 | -75.80187861772556 |
| Phenylethyl Alcohol | -53.7076053680137 |
| 2-Thiophenemethanol | -24.9648979010014 |
| Maltol | 3.4169027227750823 |
| 2-Acetylpyrrole | -12.837072044570544 |
| 4(1H)-Quinazolinone | -44.99397531607651 |
| Difurfuryl ether | -44.56887021964553 |
| 4-Hydroxy-3-methylacetophenone | -45.442214388947534 |
| Phenol | -10.375351562687147 |
| 1H-Pyrrole-2-carboxaldehyde | 20.85778135787214 |
| 4-ethyl guaiacol | -42.208403777324776 |
| 2,5-Dimethyl-4-hydroxy-3(2H)-furanone (Furaneol) | -79.86255096220098 |
| 5-Acetyldihydro-2(3H)-furanone (Solerone) | -39.779667404602826 |
| N-Methyl-2-formylpyrrol | -10.276849268450592 |
| Unknown 13 | -54.19537900283745 |
| Nonanoic acid | -16.568751968741083 |
| 2-Vinyl guaiacol | -77.21141321132494 |
| Unknown 14 | -31.642717340276803 |
| n-Decanoic acid | -38.973865597729606 |
| 2-Benzofuran-1(3H)-one | 2.594074631593448 |
| 2,3-dihydro-Benzofuran | -75.8073648729666 |
| Unknown 15 | 0.0 |
| Indole | -57.89141516797295 |
| Benzoic acid | 0.0 |
| 5-(Hydroxymethyl)dihydro-2(3H)-furanone | 47.25516154332068 |a)
b)
c)
d)
Figure S1
